# Covalent Reprogramming of Kinase Binders to Modulate Protein Abundance

**DOI:** 10.1002/advs.75153

**Published:** 2026-04-09

**Authors:** Chen Mozes, Xiaokang Jin, Miguel A. Campos, Chen Zhou, Xiaoyu Zhang

**Affiliations:** ^1^ Department of Chemistry Northwestern University Evanston USA; ^2^ Chemistry of Life Processes Institute Northwestern University Evanston USA; ^3^ Robert H. Lurie Comprehensive Cancer Center Northwestern University Chicago USA; ^4^ Center For Human Immunobiology Northwestern University Chicago USA; ^5^ International Institute for Nanotechnology Northwestern University Evanston USA

**Keywords:** enzymes, kinases, protein degraders, protein stabilizers, proteomics

## Abstract

Small molecules that modulate protein abundance through induced proximity expand the landscape beyond traditional inhibition. Here, we explore how introducing covalent or latent electrophilic groups into a multi‐kinase binder scaffold reprograms protein abundance within the kinase family. Using the broad‐spectrum kinase ligand TL13‐87 as a template, we synthesize analogs bearing α‐chloroacetamide, acrylamide, or terminal amine groups. Quantitative proteomics reveals that while most analogs have minimal global impact, MKI‐AA, a multi‐kinase inhibitor bearing an acrylamide warhead, uniquely stabilizes Aurora kinase A (AURKA). Mechanistic studies show that MKI‐AA acts post‐translationally to suppress AURKA ubiquitination and proteasomal degradation. Proteomic mapping of MKI‐AA‐induced AURKA interactors reveals changes in protein associations upon treatment, providing mechanistic insights into how MKI‐AA influences AURKA stability. Intriguingly, adding a short linker to MKI‐AA converts it from a stabilizer into a degrader, highlighting how subtle structural variations can invert functional outcomes.

## Graphical abstract

Electrophilic remodeling of a broad‐spectrum kinase binder reveals how subtle chemical changes reprogram protein fate. An acrylamide analog of a multi‐kinase binder selectively stabilizes Aurora kinase A (AURKA) by suppressing its ubiquitination, while a short‐linker variant converts this stabilizer into a degrader. These findings demonstrate that electrophilic tuning can be leveraged to bidirectionally control protein stability.

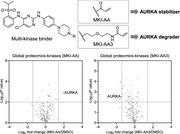

## 1 Introduction

The controlled modulation of protein abundance by small molecules has emerged as a powerful paradigm in chemical biology and drug discovery. Traditional inhibitors act by blocking enzymatic activity, yet many proteins remain functionally intractable to occupancy‐driven pharmacology [1, 2]. Targeted protein degradation (TPD) strategies, including heterobifunctional proteolysis targeting chimeras (PROTACs) and monovalent molecular glues, have demonstrated that small molecules can harness the ubiquitin‐proteasome system (UPS) to eliminate proteins of interest through induced proximity to E3 ubiquitin ligases [3, 4, 5, 6, 7]. More recently, complementary stabilization approaches such as deubiquitinase‐targeting chimeras (DUBTACs) have shown that proximity‐driven mechanisms can also rescue or enhance protein levels [8, 9, 10]. Together, these advances underscore that small molecules can be engineered not only to inhibit, but also to control the abundance of target proteins.

An emerging strategy in this area is to introduce covalent or latent electrophilic groups, such as fumarate derivatives, acrylamides, α‐chloroacetamides, vinyl sulfonylpiperazines, or terminal amines convertible to aldehydes, into existing ligands to transform them into proximity‐inducing agents. Recent studies have shown that such modifications can recruit E3 ligases, including RNF126 [11, 12], DCAF16 [13, 14, 15, 16, 17, 18, 19], DCAF11 [18], and FBXO22 [20, 21, 22], which recognize electrophilic handles through covalent engagement of reactive cysteine residues. This strategy has enabled the development of both bifunctional and glue‐like degraders that induce selective protein degradation, thereby revealing new opportunities to modulate protein abundance through induced proximity.

Building on this principle, we sought to investigate how introducing reactive groups, such as α‐chloroacetamide, acrylamide, or terminal amine, into multi‐kinase binders influences protein abundance within the kinase family. Broad‐spectrum adenosine triphosphate (ATP)‐competitive inhibitors such as TL13‐87 engage multiple kinases through conserved hinge‐binding motifs and often tolerate derivatization at the piperazine ring [23, 24]. By introducing electrophilic or terminal amine groups to these scaffolds, we hypothesize that the resulting analogs could promote proximity between specific kinase targets and endogenous effector proteins, such as E3 ligases or deubiquitinases, thereby modulating protein abundance. Such molecules provide a platform to study how covalency‐driven induced proximity shapes kinase abundance, thereby revealing potential new mechanisms of kinase regulation.

## 2 Results

### 2.1 Proteomic Characterization of Covalent and Latent Electrophilic Analogs of a Multi‐Kinase Binder

**FIGURE 1:**
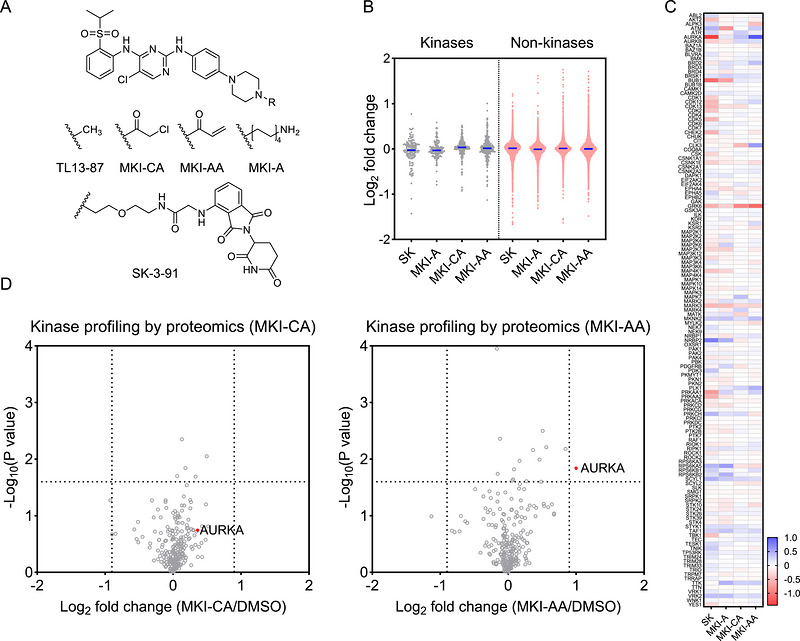
Proteomic profiling of multi‐kinase‐directed probes. (A) Chemical structures of TL13‐87, MKI‐CA, MKI‐AA, MKI‐A, and the CRBN‐based PROTAC SK‐3‐91. (B) Distribution of protein abundance changes upon probe treatment relative to DMSO in kinase and non‐kinase families. Each dot represents an individual quantified protein. The blue bars indicate the median abundance values. (C) Heatmap of relative abundance changes in quantified kinases following treatment with kinase‐directed probes. (D) Volcano plots of global kinome changes upon probe treatment in HEK293T cells (*n* = 4 biologically independent samples). *p*‐values were calculated using a two‐sided t‐test and adjusted for multiple comparisons by the Benjamini‐Hochberg method.

We selected TL13‐87, a multi‐kinase binder derived from the ALK‐selective inhibitor TAE684, by removing the 2‐methoxy group from the 2‐anilino hinge‐binding segment [24, 25] (Figure 1A). TL13‐87 has previously been used to generate heterobifunctional PROTACs through linker attachment at the piperazine ring. The resulting PROTAC shows >90% inhibition of 193 kinases in a 468‐kinase panel [24], demonstrating that it functions as a broadly active tool compound capable of engaging a large number of kinases. To assess whether introducing covalent or latent electrophilic functionalities could affect kinase abundance, we synthesized three TL13‐87 analogs bearing an α‐chloroacetamide (MKI‐CA), acrylamide (MKI‐AA), or terminal amine (MKI‐A) at the piperazine ring (Figure 1A). Using proteomics‐based whole‐proteome profiling, we evaluated the impact of these analogs on global protein and kinase abundance in HEK293T cells. For comparison, we also included a CRBN‐based PROTAC, SK‐3‐91 [23] (Figure 1A).

Global analysis of quantified kinases and non‐kinases showed that none of the compounds caused substantial proteome‐ or kinome‐wide perturbation (Figure 1B; Figure S1 and Table S1), suggesting that they may not broadly affect protein abundance through general effects on translation or the proteasome. Comparative analysis across all quantified kinases revealed that GRK6 abundance was consistently reduced by all four probes, whereas specific kinases such as AURKA and PRKAA1 were selectively down‐regulated by SK‐3‐91 (Figure 1C; Table S1). Among the TL13‐87‐derived analogs, MKI‐A weakly decreased ATM and BUB1 levels, whereas MKI‐CA had minimal impact on the abundance of all quantified kinases except for a mild reduction of GRK6 (Figure 1C,D; Figure S1). Notably, MKI‐AA selectively stabilized AURKA within the kinase family (Figure 1C,D). AURKA is a mitotic serine/threonine kinase critical for spindle assembly and centrosome maturation whose abundance is tightly regulated by proteasomal turnover [26, 27]. This observation is intriguing given that AURKA is generally destabilized upon ATP‐competitive inhibitor binding [28] or when its stabilizing cofactors, such as NEDD9, are disrupted [29]. To our knowledge, there are no prior examples of small molecules that directly stabilize AURKA, suggesting that MKI‐AA may engage an atypical binding mode or induce a proximity effect that protects AURKA from turnover.

**FIGURE 2:**
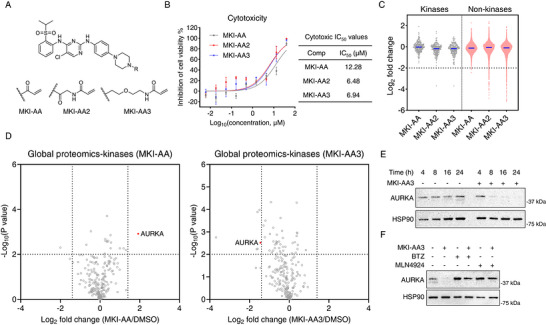
Assessing the impact of linker extension in MKI‐AA on AURKA stabilization. (A) Chemical structures of MKI‐AA, MKI‐AA2, and MKI‐AA3. (B) Dose‐response curves and IC_50_ values of MKI‐AA, MKI‐AA2, and MKI‐AA3 in HEK293T cells. Data are presented as mean ± SEM (*n* = 3). (C). Distribution of protein abundance changes upon probe treatment relative to DMSO in kinase and non‐kinase families. (D) Volcano plots of global kinome changes upon MKI‐AA or MKI‐AA3 treatment in HEK293T cells (*n* = 3 biologically independent samples for MKI‐AA3; *n* = 2 biologically independent samples for MKI‐AA). *P* values were calculated using a two‐sided t‐test and adjusted for multiple comparisons by the Benjamini‐Hochberg method. (E) Western blot analysis of AURKA in HEK293T cells following treatment with DMSO or MKI‐AA3 (1 µm) for 4–24 h. The result is representative of two experiments (*n* = 2 biologically independent samples). (F) Western blot analysis of AURKA in HEK293T cells treated with DMSO or MKI‐AA3 (1 µm, 8 h), with or without co‐treatment with BTZ (0.1 µm) or MLN4924 (1 µm). The result is representative of two experiments (*n* = 2 biologically independent samples).

We next tested whether MKI‐AA‐mediated AURKA stabilization could tolerate the introduction of an additional linker between the acrylamide and the piperazine ring. To this end, we synthesized two MKI‐AA analogs containing either a glycine (MKI‐AA2, Figure 2A) or a polyethylene glycol (PEG) spacer (MKI‐AA3, Figure 2A). Notably, incorporation of these short linkers increased cytotoxicity by approximately two‐fold in HEK293T cells (Figure 2B). Whole‐proteome analysis revealed that both MKI‐AA2 and MKI‐AA3 broadly affected protein abundance, primarily by reducing protein expression levels (Figure 2C; Figure S2 and Table S2). Interestingly, both analogs lost the ability to stabilize AURKA, and MKI‐AA3 exhibited a switch to an AURKA destabilizer (Figure 2D,E). One possible explanation for this stabilization‐to‐degradation switch is that insertion of a PEG spacer increases conformational flexibility and extends the reactive moiety into a different spatial region, enabling engagement of alternative effector proteins, such as E3 ligases, thereby promoting AURKA ubiquitination and degradation. Consistent with this hypothesis, MKI‐AA3‐induced AURKA destabilization appears to be mediated by a Cullin‐RING Ligase E3 ligase and the proteasome, as co‐treatment with the proteasome inhibitor bortezomib (BTZ) or the neddylation inhibitor MLN4924 effectively blocked this reduction (Figure 2F).

Recent studies have demonstrated that DCAF11, DCAF16, and FBXO22 are “frequent hitter” E3 ligases that can be engaged by minimal covalent warheads added to protein inhibitors [13, 16, 18, 19, 20, 21, 22, 30]. Accordingly, we examined whether any of these three ligases are required for MKI‐AA3‐mediated AURKA degradation. We evaluated MKI‐AA3 activity in wild‐type (WT) and knockout (KO) cell lines for *DCAF11* (22Rv1) [31], *DCAF16* (MDA‐MB‐231 and 22Rv1) [32, 33], and *FBXO22* (A549) [34] (Figure S3A–D). In all cases, MKI‐AA3 retained the ability to degrade AURKA in the KO cell lines, suggesting that either dependencies on these E3 ligases may emerge in additional cellular contexts not examined here or that alternative E3 ligases may be involved in this activity.

### 2.2 AURKA Stabilization is Driven by Electrophilic Warhead Reactivity Through a Post‐Translational Mechanism

**FIGURE 3:**
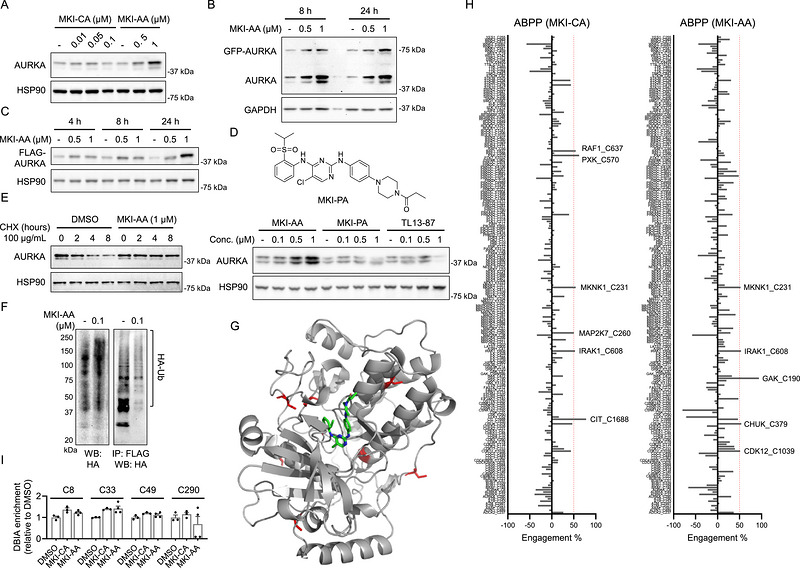
MKI‐AA stabilizes AURKA through a post‐translational mechanism. (A) Western blot analysis of AURKA in HEK293T cells following treatment with DMSO, MKI‐CA, or MKI‐AA for 24 h. The result is representative of two experiments (*n* = 2 biologically independent samples). (B) Western blot analysis of AURKA in HEK293T cells stably expressing GFP‐AURKA following treatment with DMSO or MKI‐AA for 8 or 24 h. The result is representative of two experiments (*n* = 2 biologically independent samples). (C) Western blot analysis of FLAG‐AURKA in HEK293T cells stably expressing FLAG‐AURKA following treatment with DMSO or MKI‐AA for 4, 8, or 24 h. The result is representative of two experiments (*n* = 2 biologically independent samples). (D) Top panel: chemical structure of MKI‐PA. Bottom panel: Western blot analysis of AURKA in HEK293T cells following treatment with DMSO, MKI‐AA, MKI‐PA, or TL13‐87 for 24 h. The result is representative of two experiments (*n* = 2 biologically independent samples). (E) Western blot analysis of AURKA in HEK293T cells treated with CHX for 2, 4, or 8 h, with or without co‐treatment with MKI‐AA (1 µm). The result is representative of two experiments (*n* = 2 biologically independent samples). (F) Western blot analysis of AURKA poly‐ubiquitination in HEK293T cells expressing HA‐ubiquitin and FLAG‐AURKA, with or without MKI‐AA treatment, followed by FLAG immunoprecipitation and HA blotting. The result is representative of two experiments (*n* = 2 biologically independent samples). (G) Structural model generated using the Chai‐1 showing the predicted binding pose of MKI‐AA (green) within the ATP‐binding pocket of AURKA (gray ribbon). The seven cysteine residues in AURKA are highlighted in red. (H) Target engagement (%) of quantified kinases by MKI‐CA or MKI‐AA in ABPP experiments. Data are presented as mean (*n* = 3). (I) Relative abundance of desthiobiotin iodoacetamide (DBIA)‐enriched AURKA peptides with or without treatment with MKI‐CA or MKI‐AA. Data are presented as mean ± SEM (*n* = 3).

To investigate the mechanism of MKI‐AA‐mediated AURKA stabilization, we first validated its effect on endogenous AURKA by Western blot analysis. MKI‐AA induced a dose‐dependent stabilization of AURKA in HEK293T cells, whereas the α‐chloroacetamide analog MKI‐CA showed no effect (Figure 3A). When AURKA was stably overexpressed with green fluorescent protein (GFP) or FLAG tags from heterologous promoters, MKI‐AA similarly increased its stability (Figure 3B,C). To test whether covalency is essential for this effect, we synthesized a non‐covalent analog, MKI‐PA, and also examined the parental inhibitor TL13‐87. Neither compound stabilized AURKA (Figure 3D), indicating that the covalent warhead is required for the observed stabilization. We next explored two additional MKI‐AA analogs bearing distinct warheads, including a more reactive vinyl sulfonamide (MKI‐VSA) and a less reactive, reversible cyclopropyl cyanoacrylamide (MKI‐CPA) (Figure S4A). The reversible MKI‐CPA induces dose‐dependent stabilization of AURKA, although to a lesser extent than MKI‐AA (Figure S4B). In contrast, the more reactive MKI‐VSA does not exhibit clear dose‐dependent stabilization of AURKA (Figure S4B). Collectively, across the four electrophilic warheads tested (acrylamide, cyclopropyl cyanoacrylamide, α‐chloroacetamide, and vinyl sulfonamide), the weaker electrophiles appear to confer greater AURKA stabilization. Whether this trend is directly linked to electrophilic reactivity remains an important question for future investigation.

To determine whether this effect occurs post‐translationally, we treated cells with cycloheximide (CHX) to block protein synthesis and monitored AURKA degradation over time. Co‐treatment with MKI‐AA suppressed AURKA degradation (Figure 3E), suggesting that MKI‐AA likely acts through a post‐translational stabilization mechanism. Consistent with this, we observed decreased poly‐ubiquitination of FLAG‐tagged AURKA upon MKI‐AA treatment (Figure 3F). Mechanistically, MKI‐AA likely binds directly to AURKA, as analogs of this scaffold show strong AURKA binding in kinome‐wide profiling in a previous study [24]. Molecular modeling performed using the Chai‐1 molecular modeling platform [35] supports its fit within the canonical ATP‐binding pocket of AURKA, where the acrylamide moiety extends toward the solvent‐exposed region (Figure 3G). An important question is whether the acrylamide group forms a covalent bond with a cysteine residue on AURKA, thereby acting as an intramolecular glue to modulate protein‐protein interactions and influence its turnover. However, modeling indicates that all seven cysteine residues of AURKA are distant from the MKI‐AA binding pocket (Figure 3G), arguing against direct covalent modification. This is further supported by activity‐based protein profiling (ABPP), which revealed engagement of multiple cysteines within the kinase family by MKI‐AA or MKI‐CA (Figure 3H; Figure S5 and Table S3), but no detectable engagement of the four quantified cysteines in AURKA (Figure 3I). Collectively, these results suggest that MKI‐AA‐induced AURKA stabilization likely involves the covalent engagement of another protein rather than direct modification of AURKA itself.

### 2.3 Proteomic Profiling of MKI‐AA‐Mediated AURKA Interactome

**FIGURE 4:**
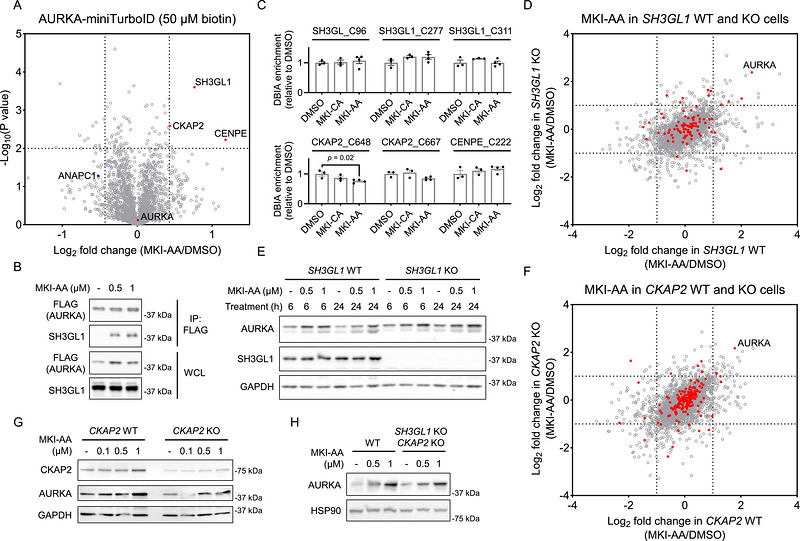
Proteomic profiling of MKI‐AA‐mediated AURKA interactome. (A) Volcano plots showing enriched biotinylated proteins comparing MKI‐AA versus DMSO treatment in HEK293T cells expressing AURKA‐miniTurbo (*n* = 2 biologically independent samples). *P* values were calculated using a two‐sided t‐test and adjusted for multiple comparisons by the Benjamini‐Hochberg method. (B) Co‐immunoprecipitation analysis showing the interaction between FLAG‐tagged AURKA and SH3GL1 with or without treatment with MKI‐AA for 2 h. The result is representative of two independent experiments (*n* = 2 biologically independent samples). (C) Relative abundance of DBIA‐enriched SH3GL1 and CKAP2 peptides with or without treatment with MKI‐CA or MKI‐AA. Data are presented as mean ± SEM (*n* = 3). (D) Global proteomic comparison of two calculated ratios, MKI‐AA/DMSO in *SH3GL1* WT cells and MKI‐AA/DMSO in *SH3GL1* KO cells, to identify proteins differentially affected by SH3GL1. Data are presented as mean (*n* = 3). (E) Western blot analysis of AURKA in HEK293T WT and *SH3GL1* KO cells following treatment with DMSO or MKI‐AA for 6 or 24 h. The result is representative of two experiments (*n* = 2 biologically independent samples). (F) Global proteomic comparison of two calculated ratios, MKI‐AA/DMSO in *CKAP2* WT cells and MKI‐AA/DMSO in *CKAP2* KO cells, to identify proteins differentially affected by CKAP2. Data are presented as mean (*n* = 3). (G) Western blot analysis of AURKA in HEK293T WT and *CKAP2* KO cells following treatment with DMSO or MKI‐AA for 24 h. The result is representative of two experiments (*n* = 2 biologically independent samples). (H) Western blot analysis of AURKA in HEK293T WT and *SH3GL1*/*CKAP2* double KO cells following treatment with DMSO or MKI‐AA for 24 h. The result is representative of two experiments (*n* = 2 biologically independent samples).

To identify MKI‐AA‐induced AURKA interactors and potential effector proteins, we used two complementary approaches. In the first approach, we performed affinity purification‐mass spectrometry (AP‐MS) under native conditions that preserve protein‐protein interactions, enriching FLAG‐tagged AURKA from HEK293T cells to compare its interactome with or without MKI‐AA treatment. In the second approach, we employed miniTurbo‐based proximity labeling [36], which allows the capture of transient or weak interactions that may be missed under native conditions. In this method, AURKA was fused to a miniTurbo tag, and biotin was added to the cell culture to label proteins in close proximity to AURKA. In both approaches, SH3GL1 emerged as the top MKI‐AA‐induced interactor of AURKA (Figure 4A; Figure S6 and Table S4). Co‐immunoprecipitation validated this finding, as FLAG‐AURKA enriched SH3GL1 only in the presence of MKI‐AA (Figure 4B). To assess whether this interaction involves covalent engagement, we re‐examined the ABPP dataset (Table S3) and found that none of the three quantified cysteines in SH3GL1 (C96, C277, and C311) showed significant engagement by MKI‐AA (Figure 4C). Nevertheless, to test whether SH3GL1 is required for AURKA stabilization, we generated *SH3GL1* KO HEK293T cells and compared AURKA abundance in WT and KO cells upon MKI‐AA treatment using both global proteomics and Western blot analysis. However, MKI‐AA stabilized AURKA to a similar extent in both WT and *SH3GL1* KO cells (Figure 4D,E; Table S5).

We next examined other potential interactors identified by proteomics. The miniTurbo‐based proximity labeling approach revealed two additional MKI‐AA‐induced neo‐interactors, CKAP2 and CENPE, that were not identified in the native AP‐MS dataset (Figure 4A; Table S4). Because *CENPE* is annotated as a common essential gene in the DepMap database [37], generating its knockout was challenging. We therefore focused on CKAP2 and generated *CKAP2* KO HEK293T cells. However, both global proteomics and Western blot analyses showed that MKI‐AA still stabilized AURKA in the *CKAP2* KO cells (Figure 4F,G; Table S6). Next, we generated *SH3GL1*/*CKAP2* double KO cells (Figure S7), and MKI‐AA‐induced AURKA stabilization persisted in the double KO cells (Figure 4H).

A previous study on branched‐chain ketoacid dehydrogenase kinase (BDK) inhibitors reported that while a thiophene analog reduced BDK protein levels, a thiazole analog switched the effect to increase BDK abundance [38]. Mechanistic studies indicated that the thiophene compound reduced BDK proximity to branched‐chain ketoacid dehydrogenase (BCKDH)‐E2, a dihydrolipoyl transacylase that serves as the core scaffold of the BCKDH complex, thereby facilitating BDK degradation. Inspired by this study, we wondered whether MKI‐AA‐mediated AURKA stabilization arises not from recruitment of a neo‐interactor but from disruption of an existing interaction partner that regulates AURKA abundance. Notably, AURKA is known to be targeted for ubiquitin‐mediated degradation by the anaphase‐promoting complex/cyclosome (APC/C), its native E3 ligase [39]. To explore whether MKI‐AA might influence this pathway, we re‐examined the miniTurbo dataset and found that one APC/C component, ANAPC1, appeared slightly disrupted upon MKI‐AA treatment (Figure 4A). This finding suggests that MKI‐AA may interfere with APC/C‐mediated recognition of AURKA, leading to reduced turnover and increased protein stabilization. However, this remains speculative, and other effector proteins or mechanisms may also contribute. For example, MKI‐AA could interfere with other components of UPS, such as alternative E3 ligases or E2 ubiquitin‐conjugating enzymes that participate in AURKA turnover, thereby reducing its ubiquitination and degradation. Another possibility is that MKI‐AA alters the subcellular localization of AURKA, which could change its local protein environment and accessibility to ubiquitination machinery. Further investigation of these potential mechanisms will be an important direction for future studies to fully elucidate how MKI‐AA modulates AURKA stability.

## 3 Discussion

Our study demonstrates that introducing electrophilic handles into multi‐kinase scaffolds can reprogram protein fate, leading to either stabilization or destabilization. The acrylamide analog MKI‐AA uniquely stabilizes AURKA through a post‐translational mechanism likely driven by induced proximity, whereas subtle structural extensions convert it into a potent degrader.

Although the precise mechanism underlying MKI‐AA‐induced AURKA stabilization remains to be fully elucidated, this probe exhibits an outcome uncommon among kinase‐directed ligands, suggesting an unexpected mechanism by which small molecules can enhance kinase stability. By stabilizing AURKA, MKI‐AA also provides a useful tool for studying AURKA‐dependent signaling. Notably, while MKI‐AA acts as a stabilizer, extending its linker by a single PEG unit converts it into a degrader. This functional switch highlights the balance between ligand‐induced stabilization and degradation driven by subtle structural modifications. Elucidating how such chemical changes invert the outcome of target engagement will be an important next step. We acknowledge that the current study is limited to cellular systems and does not yet extend to in vivo validation. Nevertheless, our findings provide mechanistic insight into induced proximity. Future studies will focus on optimizing these compounds to improve metabolic stability and pharmacological properties, thereby enabling in vivo evaluation of their stabilization and degradation activities.

## 4 Materials and Methods

### 4.1 Reagents

The horseradish peroxidase (HRP)‐linked anti‐β‐actin (clone 13E5, cat# 51255, dilution 1:5000), HRP‐linked anti‐heat‐shock protein 90 (HSP90; clone C45G5, cat# 79641, dilution 1:5000), anti‐aurora‐A (AURKA; clone D3E4Q, cat# 14475, dilution 1:1000), and HRP‐linked rabbit IgG (cat# 7074, dilution 1:1000) antibodies were purchased from Cell Signaling Technologies. The polyclonal CKAP2 antibody (cat# 25486‐1‐AP, dilution 1:1000) was purchased from Proteintech. The high‐affinity anti‐HA‐peroxidase antibody (clone 3F10, cat# 12013819001, dilution 1:5000) was purchased from Millipore Sigma. The anti‐FLAG affinity gel (clone M2, cat# A2220), anti‐FLAG HRP‐conjugated antibody (clone M2, cat# A8592, dilution 1:5000), Biotin (cat# B4639), cycloheximide (cat# C7698), and cOmplete protease inhibitor cocktail (cat#: 11873580001) were purchased from Sigma–Aldrich. Blasticidin (cat# ant‐bl‐05) and Puromycin (cat# ant‐pr‐1) were purchased from InvivoGen. Bortezomib (cat# HY‐10227) was purchased from MedChemExpress. MLN4924 (cat# 15217) was purchased from Cayman Chemical. Polyethylenimine (molecular weight 40000, cat# 24765‐1) was purchased from Polysciences. Enzyme linked chemiluminescence (ECL, cat# 32106) and ECL plus (cat# 32132) western blotting detection reagents, streptavidin agarose (cat# 20349), BCA protein assay kit (cat# 23227), anti‐SH3GL1 (polyclonal, cat# 501735533, dilution 1:1000), and tandem mass tag (TMT) isobaric label reagent (cat# 90110) were purchased from Thermo Fisher Scientific. FuGene6 (cat# E2692) transfection reagent and sequencing‐grade modified trypsin (cat# V5111) were purchased from Promega. Cas9 endonuclease was purchased from Integrated DNA Technologies (cat# 1081061).

### 4.2 Cell Lines

HEK293T, A549, MDA‐MB‐231, and 22Rv1 cells were obtained from the American Type Culture Collection (ATCC). HEK293T, MDA‐MB‐231, and A549 were cultured in DMEM (Corning, cat# 15013CV) supplemented with 10% (v/v) Fetal Bovine Serum (Omega Scientific, cat# FB‐01), 2 mm L‐glutamine (Gibco, cat# 25030081), and 100 units/mL of a 1:1 mixture of penicillin/streptomycin (Gibco, cat# 15140122). 22Rv1 cells were cultured in RPMI 1640 (Corning) with 10% (v/v) FBS (Omega Scientific) and L‐glutamine (2 mm, Gibco). All cell lines tested negative for mycoplasma contamination (ATCC, cat# 30–1012K).

### 4.3 Cloning, Mutagenesis, and Lentivirus Transduction

Human AURKA with an N‐terminal FLAG tag and human AURKA with an N‐terminal FLAG tag followed by miniTurbo were purchased as full‐length plasmids from GenScript that were cloned into the pCDH‐CMV‐MCS‐EF1‐puro vector. pHR_dSV40‐Aurora A‐GFP was obtained from Addgene (plasmid # 67924). Lentivirus was generated by co‐transfecting HEK293T cells with a plasmid containing the gene of interest, psPAX2, and pMD2.G using FuGene 6 transfection reagent. The medium containing lentiviral particles was collected after 48 h, filtered through a 0.45 µm Millex‐HV sterile syringe filter unit (MilliporeSigma), and used to transduce HEK293T cells. To generate stable cell lines, puromycin (2 µg/mL) or blasticidin (10 µg/mL) was added and incubated with the cells for 7 days.

### 4.4 Generation of *SH3GL1* and *CKAP2* Knockout Cells

HEK293T cells with *SH3GL1* and *CKAP2* CRISPR‐Cas9 knockout were generated by electroporating a complex consisting of Cas9 and three sgRNAs using the 4DNucleofector (Lonza Bioscience). The pulse code used was CM‐130 for HEK293T cells. Three sgRNAs targeting the *SH3GL1* gene (sgRNA#1: UCAACACGGUGUCCAAGAUC; sgRNA#2: GUGCAUGAUCCGCCACGGGA; sgRNA#3: ACCUGCAGCCCAACCCAGGU) were mixed for electroporation. Two sgRNAs targeting the *CKAP2* gene (sgRNA#1: GCCAAUGUUACAAUCCGGAA; sgRNA#2: CCACGAUAAGAUCCAAGCAC).

### 4.5 Generation of Transient Transfection of HA‐Ubiquitin in HEK293T Cells Stably Expressing FLAG‐AURKA

HEK293T cells stably expressing FLAG‐AURKA were grown to 50% confluency in 10 mL DMEM supplemented with 10% fetal bovine serum (FBS), penicillin, streptomycin, and glutamine in a 10 cm tissue culture dish. 3 µg of DNA was diluted in 1 mL of serum‐free DMEM, and 9 µL of PEI was added. The mixture was incubated at room temperature for 15 min and added dropwise to the cells. Cells were grown for 48 h at 37°C with 5% CO_2_ and then were split for treatment, followed by FLAG immunoprecipitation and HA blotting.

### 4.6 Cell Lysis and Western Blot

Cells were lysed in radioimmunoprecipitation lysis buffer (RIPA lysis buffer, Thermo Fisher Scientific, cat# 89900) containing 25 mm Tris‐HCl, pH 7.6, 150 nm NaCl, 1% sodium deoxycholate, 0.1% SDS, and supplemented with cOmplete protease inhibitor cocktail (Millipore Sigma, cat# 11873580001) and Pierce Universal nuclease (Thermo Fisher Scientific, cat# 88701). The suspension mixture was further probe sonicated (40% intensity, 3 rounds, 5 pulses/round). The cell lysates were then centrifuged at 16 000 g at 4°C for 10 min. Protein concentration was determined with a DC assay (BioRad, cat# 5000112). The normalized cell lysates were then mixed in with Laemmli Sample Buffer (BioRad, cat# 1610737EDU) supplemented with 10% β‐mercaptoethanol reducing agent (BioRad, cat# 1610710) and heated at 95°C for 5 min. Proteins were separated using 4%–20% Novex Tris‐Glycine mini gels (Thermo Fisher Scientific, cat# XP04205BOX). The proteins were then transferred onto a 0.2 µm polyvinylidene fluoride (PVDF) membrane (BioRad, cat# 1620177) and incubated with a solution of 5% nonfat milk in TBST buffer (0.1% Tween 20, 20 mm Tris‐HCl, pH 7.6, and 150 mm NaCl) at room temperature for 1 h. Antibodies were diluted in 5% nonfat milk in TBST buffer and applied to the membrane at dilutions listed above. After incubation, the membrane was washed three times with TBST buffer. Chemiluminescence signals of the membranes were developed using ECL western blotting detection reagent. Signals were captured using ChemiDoc MP (BioRad).

### 4.7 Cell Viability Assay

Cells were plated in a 96‐well clear‐bottom white plate (Corning) at a density of 100 000 cells per well in 200 µL of DMEM medium treated with varying concentrations of compounds for 24 h. Following treatment, 50 µL of Cell Titer Glo reagent (Promega) was added to each well and incubated for 10 min at room temperature. Luminescence was measured using a CLARIOstar Plus microplate reader (BMG Labtech).

### 4.8 Immunoprecipitations

Cell pellets were resuspended with NP‐40 lysis buffer supplemented with cOmplete protease inhibitor cocktail and Pierce Universal nuclease, followed by probe sonication (25% intensity, 2 rounds, 5 pulses/round). The cell suspensions were rotated at 4°C for 10 min before centrifugation at 16 000 g at 4°C for 10 min. Protein concentration was normalized to 2 mg/mL by a DC assay. 30 µL of whole cell lysate samples with normalized concentrations were prepared for western blot analysis by mixing with Laemmli sample buffer and heating at 95°C for 5 min. 500 µL of normalized supernatant solutions were collected for immunoprecipitation. For immunoprecipitation, 30 µL of FLAG affinity gel slurry per sample was added to the collected cell lysates and rotated at 4°C for 2 h. The affinity gel was then washed four times with immunoprecipitation wash buffer (0.2% NP‐40, 25 mM Tris‐HCl, pH 7.4, and 150 mM NaCl). The resulting slurry was then mixed in with Laemmli sample buffer and heated at 95°C for 5 min. The resulting supernatant was collected and subsequently used for Western blot analysis.

### 4.9 Global Proteomics

Cells were lysed in 100 µL of PBS with cOmplete protease inhibitor cocktail using sonication (40% intensity, 3 rounds, 5 pulses/round). Protein concentration was determined with the DC assay. A total of 100 µg of protein in 100 µL of lysis buffer was denatured with 8 m urea. For reduction, 5 µL of 200 mM dithiothreitol (DTT) stock solution in water was added, and the mixture was heated to 65 °C for 15 min. Alkylation was performed by adding 5 µL of 400 mm iodoacetamide stock solution in water and incubating in the dark at 37°C for 30 min. Samples were diluted by adding 300 µL PBS followed by 2 µg of trypsin, and digestion was carried out at 37°C for 16 h. For TMT labeling, approximately 8.5 µg of each sample in 35 µL of solution was incubated with 9 µL of acetonitrile and 5 µL of TMT tags for 1 h at room temperature. TMT labeling was quenched by adding 6 µL of a 5% solution of hydroxylamine and incubating for 15 MIN at room temperature. 2.5 µL of formic acid was added to each sample before combining all the samples. Desalting and fractionation were performed using Pierce High pH Reversed‐Phase Peptide Fractionation Kit (Thermo Fisher Scientific, Cat# 84868). In brief, the peptide sample was loaded on a spin column, desalted by washing with H_2_O with 0.1% formic acid, followed by fractionation with 30 increments of increasing gradient of acetonitrile in 10 mm NH_4_HCO_3_. Every 10th fraction was combined and concentrated, resulting in 10 distinct fractions. Peptides were analyzed by liquid chromatography‐mass spectrometry (LC‐MS) using an Orbitrap Eclipse Tribrid MS coupled with a Vanquish Neo UHPLC system. The peptides were introduced to the EASY‐Spray HPLC column (C18, 2 µm particle size, 75 µm inner diameter, and 150 mm length) and eluted at a 0.25 µL/min flow rate with the gradient: 5% buffer B (80% CH_3_CN with 0.1% FA) in buffer A (water with 0.1% FA) from 0 to 15 min, 5% to 45% buffer B from 15–155 min, and 45%–100% buffer B from 155–180 min. Voltage of the nano‐LC electrospray ionization source set to 1.5 kV. The analysis started with an MS1 master scan (Orbitrap analysis; resolution 60 000; m/z range 375–1600; RF lens 30%; standard automatic gain control (AGC) target; auto maximum injection time). For MS2 analysis, initial precursor ions were isolated by the quadrupole with an isolation window of 0.7 and then subjected to higher‐energy collisional dissociation (HCD) in the ion trap (stand AGC; collision energy 27%; maximum injection time 35 ms). After each MS2 spectrum, synchronous precursor selection (SPS) chose up to 10 MS2 fragment ions for MS3 analysis. These precursors were once again fragmented by HCD and analyzed by the Orbitrap (AGC 250%; collision energy 55%; maximum injection time 200 ms; resolution 60 000). The raw data were collected using Xcalibur (version 4.5.445.18).

Raw mass spectrometry data were analyzed using Proteome Discoverer 2.5 (Thermo Scientific). Spectra were searched with the Sequest HT algorithm against the UniProt human reference proteome (UP000005640_9606_Human.fasta). Searches were performed with fully tryptic cleavage specificity, a precursor mass tolerance of 10 ppm, and a fragment ion tolerance of 0.6 Da. Fixed modifications included carbamidomethylation of cysteines and TMT labeling on peptide N‐termini and lysine residues. Methionine oxidation and protein N‐terminal acetylation were specified as variable modifications. Peptide‐spectrum matches were evaluated using Percolator, and identifications were filtered to a 1% false‐discovery rate at both the peptide and protein levels. Reporter ion intensities were extracted from SPS‐MS3 scans with a 20 ppm integration window. Quantification was carried out using unique and razor peptides, with total peptide‐signal normalization applied across all TMT channels.

### 4.10 Cysteine‐Directed Activity‐Based Protein Profiling (ABPP)

Cells were suspended in 500 µL of PBS and lysed through probe sonification (40% intensity, 3 rounds, 5 pulses/round). The cell lysates were then centrifuged at 16 000 g at 4°C for 10 min. Protein concentration was normalized to 1 mg/mL by a DC assay. 500 µL of normalized cell lysates were labeled with 100 µm desthiobiotin iodoactamide (DBIA) by incubating for 1 h at room temperature. Protein cleanup was performed by adding 100 µL of a 1:1 mixture of hydrophobic: hydrophilic Sera‐Mag SpeedBeads to each sample. The cell lysates were incubated with the beads at room temperature for 5 min (1000 rpm rotation in a thermomixer). The lysate‐bead mixture was then incubated after adding 1 mL of absolute ethanol at room temperature for 5 min (1000 rpm rotation in a thermomixer). The supernatant was aspirated after the beads had settled using a DynaMag2 magnet. The beads were resuspended in 500 µL of a 2 m solution of urea in PBS. The proteins were then reduced with 25 µL of DTT (200 mm in HPLC‐grade water) by incubating at 65°C for 15 min. Alkylation was then performed by adding 25 µL of IA (400 mm in HPLC‐grade water) and incubating in the dark at 37°C for 30 min. The beads were washed with 1 mL of absolute ethanol three times, resuspended in 200 µL of PBS, and digested with 2 µg of trypsin at 37°C for 16 h. After digestion, the supernatant was collected and incubated with 300 µL of ABPP wash buffer (50 mM TEAB, 150 mm NaCl, 0.2% NP‐40) containing 50 µL of streptavidin agarose. The streptavidin‐peptide mixture was rotated at room temperature for 2 h. Upon completion, the beads were washed with ABPP wash buffer (1 mL × 3), PBS (1 mL × 3), and with HPLC‐grade water (1 mL × 3), in a BioSpin column. Peptides were eluted from the beads by adding 300 µL of 50% acetonitrile with 0.1% formic acid in HPLC‐grade water. The eluted peptides were dried with a SpeedVac vacuum concentrator. Samples were resuspended in 100 µL of 100 mm TEAB in 30% dry acetonitrile. For TMT labeling, added to each sample 3 µL of TMT tags and incubated for 1 h at room temperature. TMT labeling was quenched by adding 3 µL of a 5% solution of hydroxylamine and incubating for 15 min at room temperature. 5 µL of formic acid was added to each sample before combining all the samples. Desalting and fractionation were performed using the Pierce High pH Reversed‐Phase Peptide Fractionation Kit. In brief, the peptide sample was loaded on a spin column, desalted by washing with H_2_O with 0.1% formic acid, followed by fractionation with 15 increments of increasing gradient of acetonitrile in 10 mm NH_4_HCO_3_. Every fifth fraction was combined and concentrated, resulting in 5 distinct fractions. Peptides were analyzed by LC‐MS as described above.

### 4.11 Affinity Purification Mass Spectrometry (AP‐MS)

Cells were lysed in NP‐40 lysis buffer with the cOmplete protease inhibitor cocktail. After centrifugation at 16 000 g for 10 min at 4°C, the supernatant was collected for immunoprecipitation. Protein lysates were incubated with 25 µL FLAG affinity gel slurry per sample for 2 h at 4°C. The gel was washed four times in an immunoprecipitation washing buffer, followed by two washes with PBS. FLAG‐AURKA and the associated proteins were eluted by heating the beads at 65°C for 10 min in 8 m urea in PBS. The eluted proteins were then reduced with 12.5 mm DTT at 65°C for 15 min, followed by alkylation with 25 mm iodoacetamide at 37°C for 30 min. The protein solution was diluted in PBS to achieve a urea concentration of 2 m and digested with 2 µg of trypsin at 37°C for 16 h. 6 µL TMT tags were added and incubated at room temperature for 1 h, after which the reaction was quenched with 6 µL of a 5% hydroxylamine solution and 2.5 µL of formic acid. Samples were then pooled and desalted using a Sep‐Pak C18 cartridge (Waters, cat# WAT054955). The eluted peptide solution was dried with a SpeedVac concentrator and analyzed by LC‐MS as described above.

### 4.12 Proximity Labeling With miniTurbo

HEK293T cells stably expressing FLAG‐AURKA‐miniTurbo were treated with DMSO or probe in the presence of 50 µm biotin (Sigma‐Aldrich, cat# B4639) for 6 h before being collected and washed with PBS. Cell pellets were resuspended with PBS and lysed with probe sonication (40% intensity, 3 rounds, 5 pulses/round). Protein concentration was measured by the DC assay. A total of 1 mg of protein in 500 µL of lysis buffer was denatured with freshly made 8 m urea in PBS and 10 µL of 10% SDS. For reduction, 25 µL of a 200 mm DTT solution in water was added, and the sample was heated to 65°C for 15 min. Alkylation was performed by adding 25 µL of a 400 mm iodoacetamide solution in water, followed by incubation in the dark at 37°C for 30 min. Added 100 µL of 10% SDS and transferred each sample to a 15 mL tube with 5 mL PBS. For streptavidin enrichment, 50 µL streptavidin beads per sample were washed, added to each sample, and rotated for 1.5 h. The beads were then washed with 0.2% SDS/PBS (1 mL × 2), PBS (1 mL × 2), HPLC water (1 mL × 2), and 100 mm TEAB (1 mL × 1). After removing the supernatant, the beads were resuspended with 70 µL 1 m urea in 100 mm TEAB and digested with 2 µg of trypsin at 37°C for 16 h. The supernatant was collected, followed by the addition of 25 µL acetonitrile and 5 µL of TMT labels. The resulting mixture was allowed to incubate at room temperature for 1 h. TMT labeling was quenched by adding 6 µL of a 5% solution of hydroxylamine and incubating for 15 min at room temperature. 5 µL of formic acid was added to each sample before combining and drying with a SpeedVac vacuum concentrator. The pooled peptides were subjected to desalting using a Sep‐Pak C18 cartridge, and the resulting elution was dried with a SpeedVac vacuum concentrator. Peptides were analyzed by LC‐MS as described above.

### 4.13 Statistical Analysis

Quantitative data is presented as scatter plots, with the mean displayed alongside the standard error of the mean (SEM) as error bars. Comparisons between two groups were analyzed using an unpaired two‐tailed Student's *t*‐test.

## Funding

NIH R35GM154945 (X.Z.); Falk Medical Research Trust Catalyst Award (X.Z.).

## Conflicts of Interest

The authors declare that they have no conflicts of interest with the contents of this article.

## Supporting information




**Supporting file 1**: advs75153‐sup‐0001‐TableS1.xlsx

**Supporting file 2**: advs75153‐sup‐0002‐TableS2.xlsx

**Supporting file 3**: advs75153‐sup‐0003‐TableS3.xlsx

**Supporting file 4**: advs75153‐sup‐0004‐TableS4.xlsx

**Supporting file 5**: advs75153‐sup‐0005‐TableS5.xlsx

**Supporting file 6**: advs75153‐sup‐0006‐TableS6.xlsx

**Supporting file 7**: advs75153‐sup‐0007‐SuppMat.docx

## Data Availability

The mass spectrometry proteomics data have been deposited to the ProteomeXchange Consortium via the PRIDE [40] partner repository with the dataset identifier PXD071390.
