# Supplementary material for: Covalent Reprogramming of Kinase Binders to Modulate Protein Abundance
Source: Adv Sci (Weinh). 2026 Apr 9;13(36):e75153. doi: 10.1002/advs.75153 (PMC13317707; doi:10.1002/advs.75153)
Supplement: Supplementary file 7 — Supporting file 7: advs75153‐sup‐0007‐SuppMat.docx [file ADVS-13-e75153-s002.docx]

Supporting Information

Covalent Reprogramming of Kinase Binders to Modulate Protein Abundance

Chen Mozes, Xiaokang Jin, Miguel A. Campos, Chen Zhou, Xiaoyu Zhang*

C. Mozes, X. Jin, M. A. Campos, C. Zhou, X. Zhang

Department of Chemistry, Northwestern University, Evanston, United States

Chemistry of Life Processes Institute, Northwestern University, Evanston, United States

Robert H. Lurie Comprehensive Cancer Center, Northwestern University, Chicago, United States

Center for Human Immunobiology, Northwestern University, Chicago, United States

International Institute for Nanotechnology, Northwestern University, Evanston, United States
E-mail: zhang@northwestern.edu

**1. Supplementary Tables**

Supplementary Table 1. Global proteomics comparing protein expression in HEK293T cells treated with DMSO, MKI-CA, MKI-AA, MKI-A, or SK-3-91.

Supplementary Table 2. Global proteomics comparing protein expression in HEK293T cells treated with DMSO, MKI-AA, MKI-AA2, or MKI-AA3.

Supplementary Table 3. Cysteine-directed ABPP in HEK293T cells treated with DMSO, MKI-CA, or MKI-AA.

Supplementary Table 4. AP-MS and miniTurbo-based proximity labeling of the AURKA interactome in HEK293T cells treated with DMSO or MKI-AA.

Supplementary Table 5. Global proteomics comparing protein expression in HEK293T parental and *SH3GL1* KO cells treated with DMSO or MKI-AA.

Supplementary Table 6. Global proteomics comparing protein expression in HEK293T parental and *CKAP2* KO cells treated with DMSO or MKI-AA.

**2. Supplementary Figures**


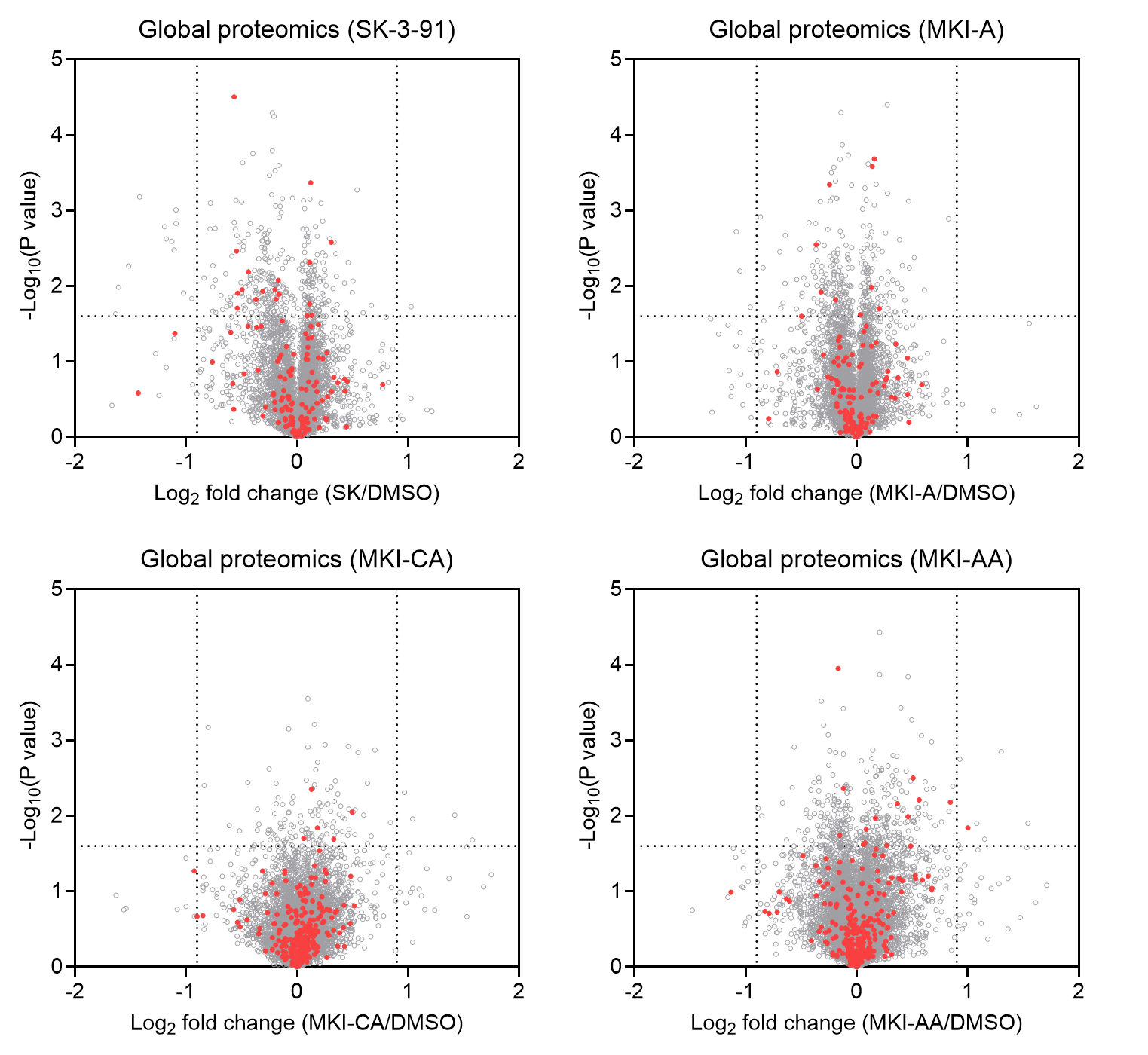


**Figure S1. Proteomic profiling of multi-kinase-directed probes.** Volcano plots showing global proteome changes upon probe treatment in HEK293T cells (n = 4 biologically independent samples for MKI-CA and MKI-AA; n = 2 biologically independent samples for MKI-A and SK-3-91). Kinases are shown in red, and non-kinase proteins in gray. *P* values were calculated using a two-sided t-test and adjusted for multiple comparisons by the Benjamini-Hochberg method.


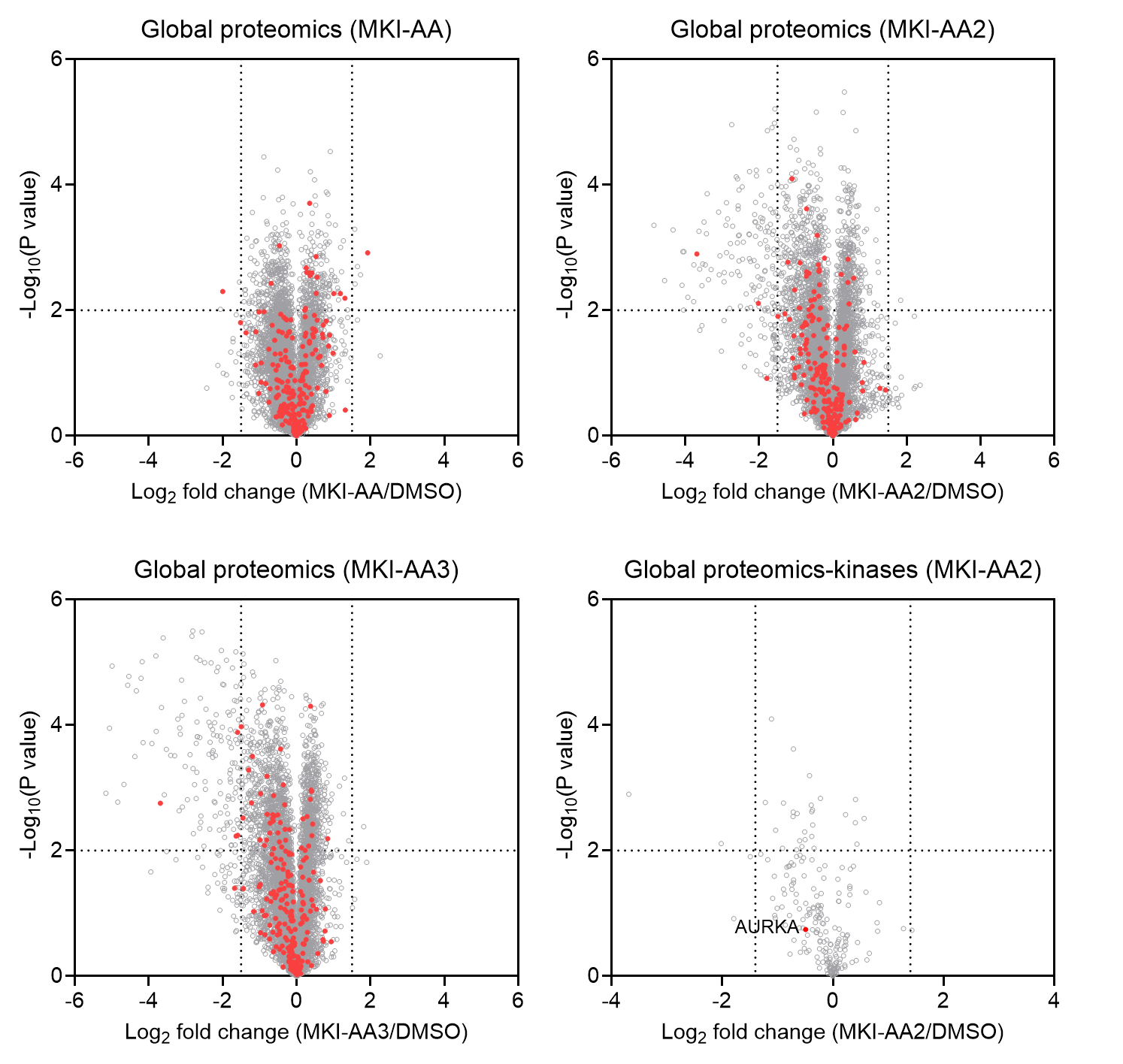


**Figure S2. Proteomic profiling of MKI-AA, MKI-AA2, and MKI-AA3.** Volcano plots showing global proteome (Kinases are shown in red, and non-kinase proteins in gray) or kinome changes upon probe treatment in HEK293T (n = 3 biologically independent samples for MKI-AA3; n = 2 biologically independent samples for MKI-AA and MKI-AA2). *P* values were calculated using a two-sided t-test and adjusted for multiple comparisons by the Benjamini-Hochberg method.

**
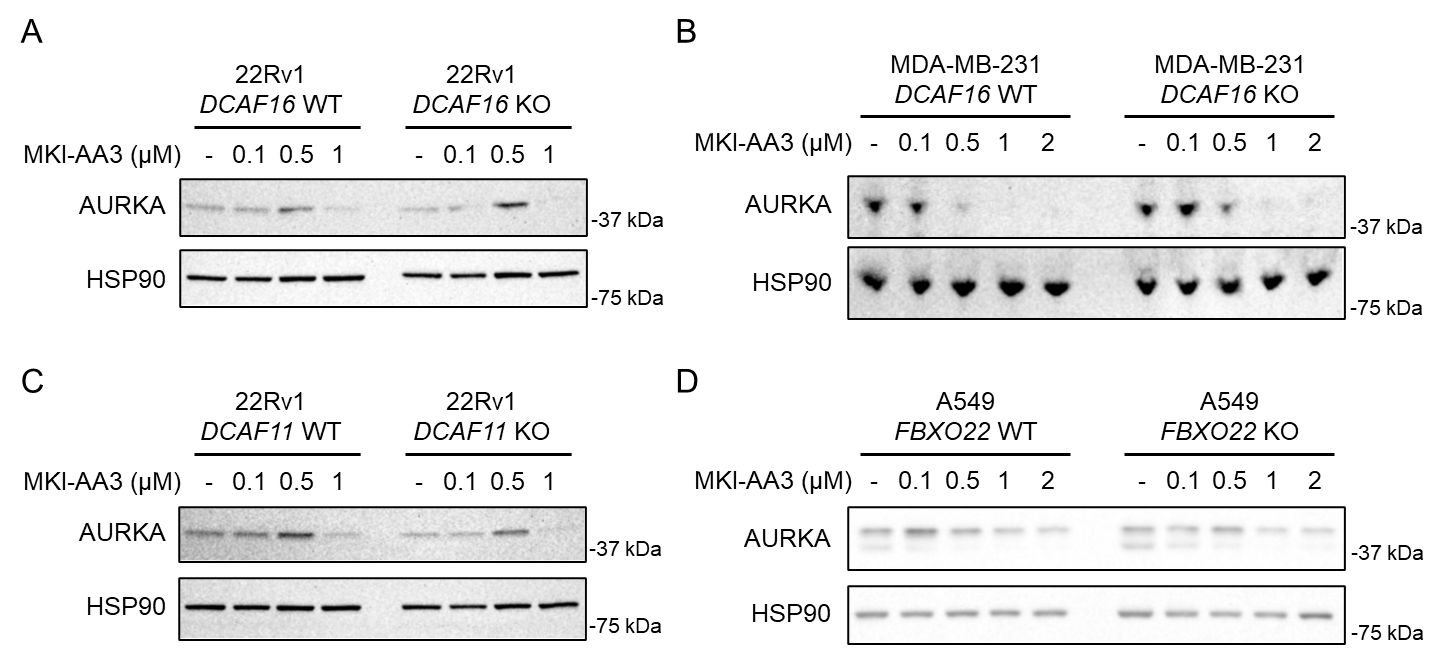
**

**Figure S3. Assessing the dependence of DCAF11, DCAF16, and FBXO22 on MKI-AA3-mediated AURKA degradation. A**. Western blot analysis of AURKA in 22Rv1 parental (*DCAF16* WT) and *DCAF16* KO cells following treatment with DMSO or MKI-AA3 for 24 hours. The result is representative of two experiments (n = 2 biologically independent samples). **B**. Western blot analysis of AURKA in MDA-MB-231 parental (*DCAF16* WT) and *DCAF16* KO cells following treatment with DMSO or MKI-AA3 for 24 hours. The result is representative of two experiments (n = 2 biologically independent samples). **C**. Western blot analysis of AURKA in 22Rv1 parental (*DCAF11* WT) and *DCAF11* KO cells following treatment with DMSO or MKI-AA3 for 24 hours. The result is representative of two experiments (n = 2 biologically independent samples). **D**. Western blot analysis of AURKA in A549 parental (*FBXO22* WT) and *FBXO22* KO cells following treatment with DMSO or MKI-AA3 for 24 hours. The result is representative of two experiments (n = 2 biologically independent samples).


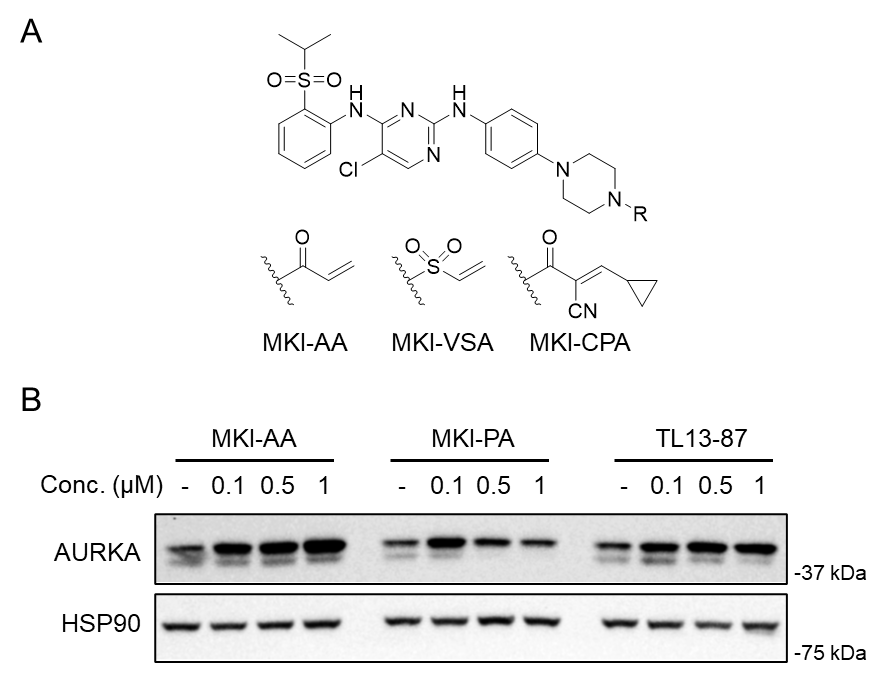


**Figure S4. Evaluation of different MKI warheads on AURKA stabilization. A**. Chemical structures of MKI-AA, MKI-vinyl sulfonamide (VSA), and MKI-cyclopropyl cyanoacrylamide (CPA). **B**. Western blot analysis of AURKA in HEK293T cells following treatment with DMSO, MKI-AA, MKI-VSA, or MKI-CPA for 24 hours. The result is representative of two experiments (n = 2 biologically independent samples).


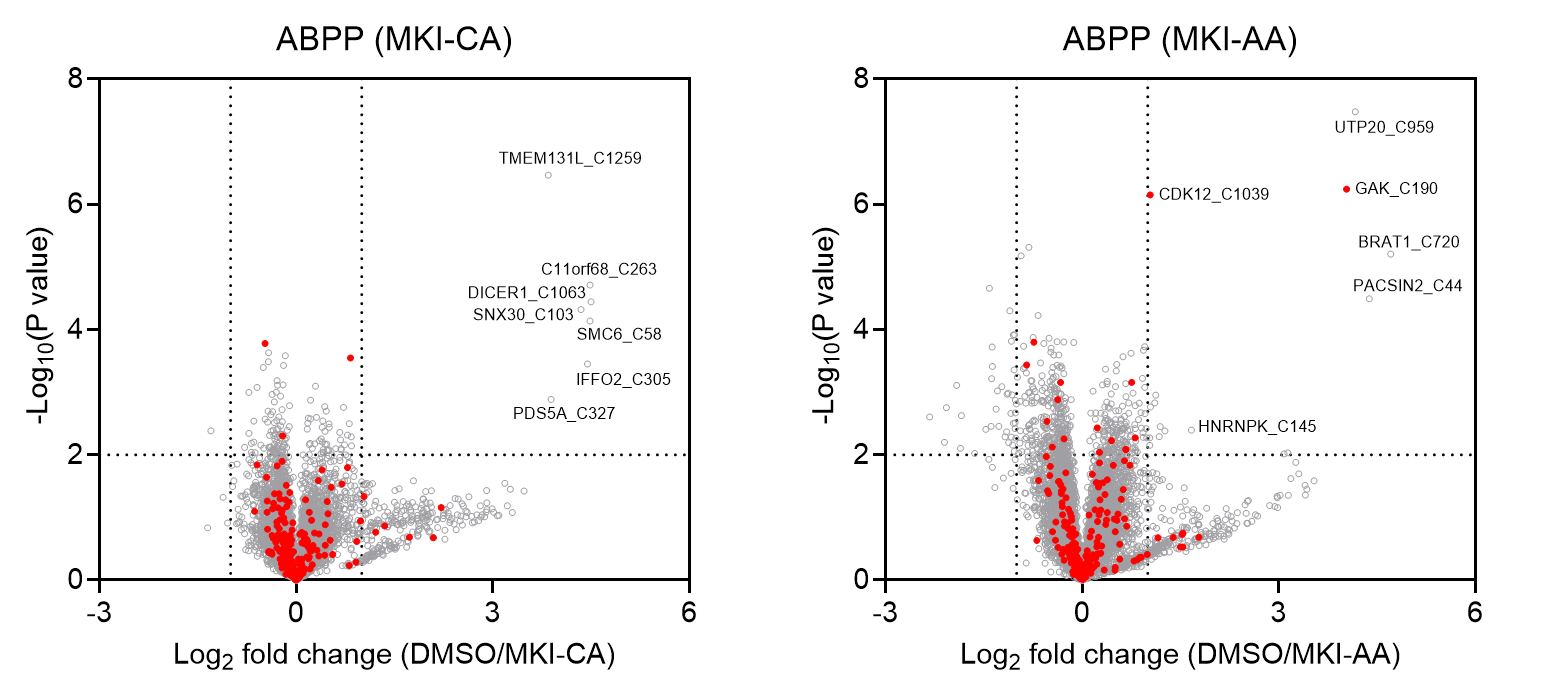


**Figure S5. ABPP analysis of MKI-CA and MKI-AA target engagement.** Volcano plots show global cysteine engagement by MKI-CA or MKI-AA. Kinases are shown in red and non-kinase proteins in gray (n = 3 biologically independent samples). HEK293T cells were treated with MKI-CA (0.2 µM) or MKI-AA (2 µM) for 2 hours. *P* values were calculated using a two-sided t-test and adjusted for multiple comparisons by the Benjamini-Hochberg method.

**Figure S6. AP-MS profiling of MKI-AA-mediated AURKA interactome.** Volcano plots showing enrichment of FLAG-AURKA-associated proteins comparing MKI-AA versus DMSO treatment in HEK293T cells (n = 3 biologically independent samples). *P* values were calculated using a two-sided t-test and adjusted for multiple comparisons by the Benjamini-Hochberg method.


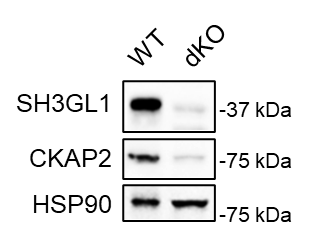


**Figure S7.** Western blot analysis of SH3GL1 and CKAP2 in HEK293T *SH3GL1*/*CKAP2* double KO cells. The result is representative of two experiments (n = 2 biologically independent samples).

**3. Synthetic Procedures**

All reactions were carried out under a nitrogen atmosphere in flamed-dried glassware with magnetic stirring unless stated otherwise. Chemicals and reagents were purchased from a variety of vendors, including Sigma Aldrich, Thermo Fisher Scientific, Ambeed, CombiBlocks, and were used without further purification, unless noted otherwise. Anhydrous solvents were obtained as commercially available pre-dried, oxygen-free formulations. Normal and reverse phase purification of reaction products was carried out by flash chromatography on Biotage Selekt systems with ultragrade silica cartridges. Preparative thin layer chromatography (PTLC) was carried out using glass backed PTLC 20x20 cm plates 250 or 500 μm thickness (Miles Scientific). Analytical thin-layer chromatography was performed on 0.25 mm silica gel 60-F plates. Visualization was done with UV light and/or by ninhydrin or KMnO_4_ staining. ^1^H-NMR spectra were recorded on a Bruker AVANCE III 500 MHz with DCH Cryoprobe (500 MHz) spectrometer and are reported in ppm using solvent as an internal standard (CDCl_3_ at 7.26 ppm, CDCl3 with 0.03% v/v TMS at 7.26 ppm, MeOD at 3.31 ppm). Data are reported as (bs = broad singlet, s = singlet, d = doublet, t = triplet, q = quartet, m = multiplet, etc.; coupling constant(s) in Hz; integration) Proton-decoupled ^13^C NMR spectra were recorded on Bruker AVANCE III 500 MHz with DCH Cryoprobe (126 MHz) spectrometer and are reported in ppm using residual solvent as an internal standard (CDCl_3_ at 7.26 ppm, CDCl_3_ with 0.03% v/v TMS at 7.26 ppm, MeOD at 3.31 ppm). Low-resolution mass spectra (not reported herein) were obtained on a WATERS Acquity I-Class UPLC-MS with 17 a single quad detector (ESI), ELSD, and PDA. High resolution mass spectra were obtained using an Agilent 6201 MSLC-TOF (ESI).

**5-chloro-N4-(2-(isopropylsulfonyl)phenyl)-N2-(4-(piperazin-1- yl)phe-nyl)pyrimidin-e-2,4-diamine** (**MKI-NH**)

The synthesis was performed according to a previously reported procedure.^[1]^ ^1^H-NMR (500 MHz, MeOD): δ 8.49 (d, *J* = 8.0 Hz, 1H), 8.14 (s, 1H), 7.92 (dd, *J* = 8.0, 1.5 Hz, 1H), 7.68 (dt, *J* = 9.0, 1.5 Hz, 1H), 7.42-7.39 (m, 3H), 6.99 (d, *J* = 9.0 Hz, 2H), 3.39 (s, 8H), 3.37-3.32 (m, 1H), 1.24 (d, *J* = 7.0 Hz, 6H). ^13^C-NMR (126 MHz, MeOD): δ 157.92, 157.22, 150.86, 148.63, 138.77, 135.98, 133.22, 132.47, 127.51, 126.17, 125.83, 124.49, 118.64, 106.60, 56.93, 44.87, 15.45.

**1-(4-(4-((5-chloro-4-((2-(isopropylsulfonyl)phenyl)amino)pyrimidin-2-yl)amino)phenyl)piperazin-1-yl)prop-2-en-1-one (MKI-AA)**

A solution of acrylic acid (3.3 μL, 0.048 mmol, 1.2 equiv.) and propanephosphonic acid anhydride (T3P) (43 mg, 0.051 mmol, 1.7 equiv.) in dry DMF (2 mL) under N_2_ was cooled to 0 °C and stirred for 10 minutes. Then diisopropylethylamine (20.9 µL, 0.12 mmol, 3 equiv.) was added followed by amine MKI-NH (20 mg, 0.04 mmol, 1 equiv.) and the mixture was stirred at 0 °C for another 30 min, then at room temperature for overnight. The reaction mixture was concentrated under reduced pressure and purified by preparative TLC (hexane/ethyl acetate 1:4) to yield **MKI-AA** as a pale yellow solid (10 mg, 18.5 μmol, 46%). ^1^H-NMR (500 MHz, CDCl_3_): δ 9.63 (s, 1H), 8.58 (d, *J* = 8.5 Hz, 1H), 8.10 (s, 1H), 7.90 (dd, *J* = 8.0, 1.5 Hz, 1H), 7.56 (t, *J* = 7.0 Hz, 1H), 7.42 (d, *J* = 9.0 Hz, 2H), 7.24 (t, *J* = 8.0 Hz, 2H), 6.90 (d, *J* = 8.5 Hz, 2H), 6.61 (dd, *J* = 17.0, 10.5 Hz, 1H), 6.33 (dd, *J* = 17.0, 2.0 Hz, 1H), 5.74 (dd, *J* = 10.5, 2.0 Hz, 1H), 3.87 (bs, 2H), 3.74 (bs, 2H), 3.26-3.20 (m, 1H), 3.15 (t, *J* = 5.5 Hz, 4H), 1.31 (d, *J* = 7.0 Hz, 6H). ^13^C-NMR (126 MHz, CDCl_3_): δ 165.54, 158.06, 155.56, 155.06, 147.31, 138.54, 134.54, 132.60, 131.40, 128.39, 127.41, 124.63, 123.60, 123.27, 122.08, 117.58, 105.99, 55.76, 50.70, 50.17, 45.92, 42.04, 15.49. HRMS (ESI+) m/z calculated for C_26_H_29_ClN_6_O_3_S [M+H]^+^: 541.1783; found: 541.1766.

**2-chloro-1-(4-(4-((5-chloro-4-((2-(isopropylsulfonyl)phenyl)amino)pyrimidin-2-yl)amino)phenyl)piperazin-1-yl)ethenone (MKI-CA)**

This compound was synthesized with the same procedure as MKI-AA, starting from chloroacetic acid (2.9 μL, 0.048 mmol, 1.2 equiv.). The preparative TLC was run with hexane/ethyl acetate 1:4 to yield **MKI-CA** as a yellow solid (7.1 mg, 12.6 μmol, 31%). ^1^H-NMR (500 MHz, CDCl_3_): δ 9.95 (s, 1H), 8.58 (d, *J* = 8.5 Hz, 1H), 8.11 (s, 1H), 7.90 (dd, *J* = 8.0, 1.5 Hz, 1H), 7.57 (t, *J* = 7.0 Hz, 1H), 7.42 (d, *J* = 9.0 Hz, 2H), 7.25 (t, *J* = 8.5 Hz, 2H), 7.10 (bs, 1H), 6.91 (d, *J* = 9.0 Hz, 2H), 4.12 (s, 2H), 3.81 (m, 2H), 3.71 (m, 2H), 3.28-3.22 (m, 1H), 3.20 (m, 2H), 3.15 (m, 2H), 1.31 (d, *J* = 7.0 Hz, 6H). ^13^C-NMR (126 MHz, CDCl_3_): δ 165.28, 157.89, 155.58, 154.83, 147.20, 138.51, 134.55, 132.67, 131.44, 124.68, 123.57, 123.34, 122.08, 117.73, 106.17, 55.79, 50.51, 50.09, 46.44, 42.27, 40.95, 15.50. HRMS (ESI+) m/z calculated for C_25_H_28_Cl_2_N_6_O_3_S [M+H]^+^: 563.1393; found: 563.1406.

**2-(4-(4-((5-chloro-4-((2-(isopropylsulfonyl)phenyl)amino)pyrimidin-2-yl)amino)phenyl)piperazine-1-carbonyl)-3-cyclopropylacrylonitrile (MKI-CPA)**

This compound was synthesized with the same procedure as MKI-AA, starting from 2-cyano-3-cyclopropyl-2-propenoic acid (8.2 mg, 0.06 mmol, 1 equiv.). The preparative TLC was run with hexane/ethyl acetate 1:4 to yield **MKI-CPA** as a yellow solid (10.7 mg, 17.6 μmol, 29%). ^1^H-NMR (500 MHz, CDCl_3_): δ 9.76 (s, 1H), 8.55 (d, *J* = 8.5 Hz, 1H), 8.08 (s, 1H), 7.90 (dd, *J* = 8.0, 1.5 Hz, 1H), 7.56 (t, *J* = 7.0 Hz, 1H), 7.44-7.40 (m, 2H), 7.27 (t, *J* = 7.5 Hz, 2H), 6.91 (d, *J* = 9.0 Hz, 2H), 6.66 (d, *J* = 11.5 Hz, 1H), 3.88-3.77 (m, 4H), 3.26-3.14 (m, 5H), 2.14-2.06 (m, 1H), 1.31 (d, *J* = 7.0 Hz, 6H), 1.28-1.23 (m, 2H), 0.93-0.89 (m, 2H),. ^13^C-NMR (126 MHz, CDCl_3_): δ 166.65, 162.36, 157.22, 155.75, 153.22, 147.30, 138.19, 134.59, 132.20, 131.46, 124.83, 123.66, 122.29, 117.75, 117.61, 115.75, 107.38, 106.04, 55.88, 50.14, 15.83, 15.48, 10.92, 10.50. HRMS (ESI+) m/z calculated for C_30_H_33_ClN_7_O_3_S [M+H]^+^: 606.2049; found: 606.2041.

**5-chloro-N4-(2-(isopropylsulfonyl)phenyl)-N2-(4-(4-(vinylsulfonyl)piperazin-1-yl)phenyl)pyrimidine-2,4-diamine (MKI-VSA)**

A solution of MKI-NH (30 mg, 0.06 mmol, 1 equiv.) in DCM (1 mL) was cooled to 0 °C before adding triethylamine (29 μL, 0.21 mmol, 3.5 equiv.) and ethenesulfonyl chloride (9.1 mg, 0.07 mmol, 1.2 equiv.) dropwise. The mixture was stirred for 5 minutes at 0 °C and another 20 minutes at room temperature. The reaction mixture was concentrated under reduced pressure and purified by preparative TLC (hexane/ethyl acetate 1:1) to yield **MKI-VSA** as a pale yellow solid (4.4 mg, 7.6 μmol, 13%). ^1^H-NMR (500 MHz, CDCl_3_): δ 9.77 (s, 1H), 8.55 (d, *J* = 8.5 Hz, 1H), 8.08 (s, 1H), 7.91 (dd, *J* = 8.0, 1.5 Hz, 1H), 7.56 (t, *J* = 7.5 Hz, 1H), 7.42 (d, *J* = 9.5 Hz, 2H), 7.27 (t, *J* = 7.5 Hz, 2H), 6.90 (d, *J* = 9.0 Hz, 2H), 6.47 (dd, *J* = 16.5, 10.0 Hz, 1H), 6.30 (d, *J* = 16.5 Hz, 1H), 6.10 (d, *J* = 10.0 Hz, 1H), 3.36-3.33 (m, 4H), 3.25-3.18 (m, 5H), 1.31 (d, *J* = 7.0 Hz, 6H). ^13^C-NMR (126 MHz, CDCl_3_): δ 157.17, 155.81, 153.11, 147.27, 138.20, 134.57, 132.32, 131.51, 129.41, 124.89, 123.71, 123.65, 122.36, 122.23, 117.82, 106.14, 55.91, 50.08, 45.73, 15.50. HRMS (ESI+) m/z calculated for C_25_H_30_ClN_6_O_4_S_2_ [M+H]^+^: 577.1453; found: 577.1451.

**1-(4-(4-((5-chloro-4-((2-(isopropylsulfonyl)phenyl)amino)pyrimidin-2-yl)amino)phenyl)piperazin-1-yl)propan-1-one (MKI-PA)**

This compound was synthesized with the same procedure as MKI-AA, starting from propionic acid (3.8 μL, 0.048 mmol, 1.2 equiv.). The preparative TLC was run with hexane/ethyl acetate 1:4 to yield **MKI-PA** as a brown solid (13 mg, 24 μmol, 60%). ^1^H-NMR (500 MHz, CDCl_3_): 9.63 (s, 1H), 8.58 (d, *J* = 8.5 Hz, 1H), 8.09 (s, 1H), 7.90 (dd, *J* = 8.0, 1.5 Hz, 1H), 7.55 (dt, *J* = 8.0, 1.5 Hz, 1H), 7.42 (d, *J* = 9.0 Hz, 2H), 7.35 (bs, 1H), 7.24 (dt, *J* = 7.5, 1.0 Hz, 1H), 6.90 (d, *J* = 9.0 Hz, 2H), 3.81-3.78 (m, 2H), 3.65-3.62 (m, 2H), 3.27-3.18 (m, 1H), 3.14-3.09 (m, 4H), 2.40 (q, *J* = 7.5 Hz, 2H), 1.31 (d, *J* = 7.0 Hz, 6H), 1.18 (t, *J* = 7.5 Hz, 3H). ^13^C-NMR (126 MHz, CDCl_3_): δ 172.49, 158.03, 155.57, 154.93, 147.40, 138.52, 134.54, 132.53, 131.39, 124.63, 123.62, 123.28, 122.09, 117.53, 105.93, 55.76, 50.60, 50.24, 45.52, 41.65, 26.62, 15.48, 9.62. HRMS (ESI+) m/z calculated for C_26_H_31_ClN_6_O_3_S [M+H]^+^: 543.1940; found: 543.1936.

**tert-butyl-(8-(4-(4-((5-chloro-4-((2-(isopropylsulfonyl)phenyl)amino)pyrimidin-2-yl)amino)phenyl)piperazin-1-yl)octyl)carbamate (MKI-A-Boc)**

MKI-NH (100 mg, 0.17 mmol, 1 equiv.), N-Boc-8-bromooctane-amine (63 mg, 0.2 mmol, 1.2 equiv.) and potassium carbonate (71 mg, 0.5 mmol, 3 equiv.) were dissolved with 2.5 acetonitrile and refluxed at 80 °C for overnight. The mixture was diluted with dichloromethane and filtered. The filtrate was concentrated under reduced pressure and purified by flash chromatography (10%-100% EtOAc in hexane) to yield **MKI-A-Boc** as an off-white solid (54 mg, 76 μmol, 44%). ^1^H-NMR (500 MHz, CDCl_3_): δ 9.61 (s, 1H), 8.59 (d, *J* = 8.0 Hz, 1H), 8.10 (s, 1H), 7.88 (d, *J* = 7.5 Hz, 1H), 7.54 (t, *J* = 8.0 Hz, 1H), 7.37 (d, *J* = 7.0 Hz, 2H), 7.22 (t, *J* = 7.5 Hz, 1H), 6.92-6.89 (m, 3H), 4.50 (bs, 1H), 3.26-3.21 (m, 1H), 3.20-3.14 (m, 4H), 3.13-3.08 (m, 2H), 2.63-2.60 (m 4H), 2.41-2.37 (m, 2H), 1.55-1.50 (m, 2H), 1.44 (s, 9H), 1.31 (s, 10H), 1.30 (s, 6H). ^13^C-NMR (126 MHz, CDCl_3_): δ 158.23, 155.99, 155.34, 155.30, 147.92, 138.53, 134.48, 131.44, 131.20, 124.30, 123.41, 122.96, 122.27, 116.66, 105.75, 79.03, 58.83, 55.58, 53.33, 49.79, 40.63, 30.08, 29.50, 29.23, 28.45, 27.54, 26.92, 26.76, 15.37.

**N2-(4-(4-(8-aminooctyl)piperazin-1-yl)phenyl)-5-chloro-N4-(2-(isopropylsulfonyl)phenyl)pyrimidine-2,4-diamine (MKI-A)**

MKI-A-Boc (54 mg, 0.076 mmol, 1 equiv.) was dissolved with DCM (240 μL) followed by a dropwise addition of TFA (60 µL). The mixture was stirred overnight at room temperature and then concentrated under reduced pressure. The crude mixture was purified with reverse-phase chromatography on a Biotage C18 column (acetonitrile in H_2_O, 5-95% gradient) to afford **MKI-A** as a yellow solid (10 mg, 16 μmol, 21%). ^1^H-NMR (500 MHz, MeOD): δ 8.54-8.45 (m, 1H), 8.14 (s, 1H), 7.92 (d, *J* = 8.0 Hz, 1H), 7.68 (t, *J* = 7.5 Hz, 1H), 7.44-7.37 (m, 3H), 6.99 (d, *J* = 9.0 Hz, 2H), 3.84-3.75 (m, 2H), 3.72-3.65 (m, 2H), 3.37-3.32 (m, 1H), 3.28-3.23 (m, 2H), 3.22-3.18 (m, 2H), 3.12-3.03 (m, 2H), 2.92 (t, *J* = 7.5 Hz, 2H), 1.84-1.77 (m, 2H), 1.70-1.63 (m, 2H), 1.46-1.40 (m, 8H), 1.24 (d, *J* = 7.5 Hz, 6H). ^13^C-NMR (126 MHz, MeOD): δ 158.08, 156.77, 149.97, 148.31, 138.58, 136.02, 132.89, 132.48, 127.67, 126.29, 126.05, 124.68, 118.51, 106.61, 58.01, 56.95, 53.10, 48.35, 40.69, 29.95, 28.54, 27.48, 27.32, 25.00, 15.44. HRMS (ESI+) m/z calculated for C_31_H_45_ClN_7_O_2_S+ [M+H]^+^: 614.3038, found: 614.3050.

**tert-butyl (2-(4-(4-((5-chloro-4-((2-(isopropylsulfonyl)phenyl)amino)pyrimidin-2-yl)amino)phenyl)piperazin-1-yl)-2-oxoethyl)carbamate (MKI-Gly-Boc)**

Boc-Gly-OH (17.5 mg, 0.1 mmol, 1 equiv.), propanephosphonic acid anhydride (T3P) (108 mg, 0.17mmol, 1.7 equiv.) and diisopropylethylamine (52.3 µL, 0.3 mmol, 3 equiv.) were dissolved with dry DMF (3 mL) under N_2_ followed by addition of MKI-NH (50 mg, 0.1 mmol, 1 equiv.) and stirred overnight. The reaction mixture was concentrated under reduced pressure and purified by preparative TLC (MeOH:DCM 1:9) to yield **MKI-Gly-Boc** as a yellow solid (36.6 mg, 57 μmol, 57%). ^1^H-NMR (500 MHz, CDCl_3_): δ 9.63 (s, 1H), 8.58 (d, *J* = 8.5 Hz, 1H), 8.10 (s, 1H), 7.89 (dd, *J* = 8.0, 2.0 Hz, 1H), 7.55 (t, *J* = 8.5 Hz, 1H), 7.41 (d, *J* = 9.0 Hz, 2H), 7.27 (bs, 1H), 7.24 (t, *J* = 8.5 Hz, 2H), 6.88 (d, *J* = 9.0 Hz, 2H), 5.60 (s, 1H), 4.01 (d, *J* = 4.5 Hz, 2H), 3.81-3.79 (m, 2H), 3.57-3.55 (m, 2H), 3.25-3.20 (m, 1H), 3.14-3.10 (m, 4H), 1.45 (s, 9H), 1.30 (d, *J* = 6.5 Hz, 6H). ^13^C-NMR (126 MHz, CDCl_3_): δ 167.06, 157.96, 155.99, 155.50, 154.98, 147.11, 138.50, 134.52, 132.76, 131.38, 124.57, 123.53, 123.26, 122.04, 117.67, 106.01, 79.87, 55.74, 50.36, 50.14, 44.50, 42.35, 42.07, 28.48, 15.46.

**2-amino-1-(4-(4-((5-chloro-4-((2-(isopropylsulfonyl)phenyl)amino)pyrimidin-2-yl)amino)phenyl)piperazin-1-yl)ethenone (MKI-Gly)**

MKI-Gly-Boc (60 mg, 0.093 mmol, 1 equiv.) was dissolved with DCM (1.5 mL) followed by a dropwise addition of TFA (375 µL). The mixture was stirred for 2h at room temperature and then concentrated under reduced pressure. The crude mixture was purified with reverse-phase chromatography on a Biotage C18 column (acetonitrile in H_2_O, 5-95% gradient) to afford **MKI-Gly** as a yellow solid (16.7 mg, 30.6 μmol, 32%). ^1^H-NMR (500 MHz, MeOD): δ 8.54 (d, *J* = 8.0 Hz, 1H), 8.11 (s, 1H), 7.90 (dd, *J* = 8.0, 2.0 Hz, 1H), 7.66 (dt, *J* = 9.0, 2.0 Hz, 1H), 7.40-7.35 (m, 3H), 6.98 (d, *J* = 8.5 Hz, 2H), 4.01 (s, 2H), 3.81-3.78 (m, 2H), 3.63-3.60 (m, 2H), 3.35-3.31 (m, 1H), 3.23-3.14 (m, 4H), 1.24 (d, *J* = 7.0 Hz, 6H). ^13^C-NMR (126 MHz, MeOD): δ 165.63, 163.40, 157.47, 152.49, 148.78, 139.08, 135.97, 133.40, 132.38, 126.84, 125.76, 125.34, 124.13, 118.59, 106.39, 56.93, 51.29, 51.02, 45.60, 43.16, 40.95, 15.46.

**N-(2-(4-(4-((5-chloro-4-((2-(isopropylsulfonyl)phenyl)amino)pyrimidin-2-yl)amino)phenyl)piperazin-1-yl)-2-oxoethyl)acrylamide (MKI-AA2)**

This compound was synthesized with the same procedure as MKI-AA, using acrylic acid (2.5 μL, 0.036 mmol, 1.2 equiv.), T3P (32 mg, 0.051 mmol, 1.7 equiv.), DIPEA (16 μL, 0.09 mmol, 3 equiv.), MKI-Gly (16.7 mg, 0.03 mmol, 1 equiv.) and DMF (2 mL). The preparative TLC was run with MeOH/DCM 1:9 to yield **MKI-AA2** as a yellow solid (6.0 mg, 1.67 μmol, 33%). ^1^H-NMR (500 MHz, CDCl_3_): 9.65 (s, 1H), 8.58 (d, *J* = 8.5 Hz, 1H), 8.10 (s, 1H), 7.90 (dd, *J* = 8.0, 2.0 Hz, 1H), 7.56 (t, *J* = 8.5 Hz, 1H), 7.43 (d, *J* = 9.0 Hz, 2H), 7.25 (t, *J* = 8.0 Hz, 1H), 7.14 (bs, 1H), 6.90 (d, *J* = 8.5 Hz, 2H), 6.77 (bs, 1H), 6.32 (dd, *J* = 17.0, 1.5 Hz, 1H), 6.21 (dd, *J* = 17.0, 10.0 Hz, 1H), 5.69 (dd, *J* = 10.5, 1.5 Hz, 1H), 4.20 (d, *J* = 4.5 Hz, 2H), 3.84-3.81 (m, 2H), 3.63-3.60 (m, 2H), 3.28-3.19 (m, 1H), 3.17-3.12 (m, 4H), 3.31 (d, *J* = 7.0 Hz, 6H). ^13^C-NMR (126 MHz, CDCl_3_): δ 166.51, 165.55, 157.89, 155.57, 154.88, 147.10, 138.51, 134.53, 132.80, 131.43, 130.50, 127.05, 124.67, 123.56, 123.33, 122.04, 117.78, 106.17, 55.78, 50.43, 50.18, 44.60, 42.22, 41.42, 15.49. HRMS (ESI+) m/z calculated for C_28_H_32_ClN_7_O_4_S [M+H]^+^: 598.1998; found: 598.1987.

**tert-butyl (2-(2-(4-(4-((5-chloro-4-((2-(isopropylsulfonyl)phenyl)amino)pyrimidin-2-yl)amino)phenyl)piperazin-1-yl)ethoxy)ethyl)carbamate (MKI-PEG1-Boc)**

MKI-NH (100 mg, 0.17 mmol, 1 equiv.), N-Boc-PEG1-bromide (66 mg, 0.2 mmol, 1.2 equiv.) and potassium carbonate (83 mg, 0.5 mmol, 3 equiv.) were dissolved with 2.5 acetonitrile and refluxed at 80 °C for overnight. The mixture was diluted with dichloromethane and filtered. The filtrate was concentrated under reduced pressure and purified by preparative TLC (MeOH:DCM 1:9) to yield **MKI-PEG1-Boc** as a yellow solid (82.7 mg, 122 μmol, 61%). ^1^H-NMR (500 MHz, CDCl_3_): δ 9.60 (s, 1H), 8.59 (d, *J* = 8.5 Hz, 1H), 8.09 (s, 1H), 7.87 (d, *J* = 8.0 Hz, 1H), 7.53 (t, *J* = 7.5 Hz, 1H) 7.36 (d, *J* = 8.5 Hz, 2H), 7.20 (t, *J* = 7.5 Hz, 2H), 6.88 (d, *J* = 8.5 Hz, 2H), 5.23 (bs, 1H), 3.62 (t, *J* = 6.0 Hz, 2H), 3.53 (t, *J* = 5.0, 2H), 3.33-3.29 (m, 2H), 3.25-3.20 (m, 1H), 3.20-3.18 (m, 4H), 2.69-2.67 (m, 4H), 2.64 (t, *J* = 5.5 Hz, 2H), 1.42 (s, 9H), 1.29 (d, *J* = 7.0 Hz, 6H). ^13^C-NMR (126 MHz, CDCl_3_): δ 158.34, 156.13, 155.43, 155.32, 147.84, 138.62, 134.56, 131.68, 131.28, 124.35, 123.50, 123.05, 122.34, 116.73, 105.73, 79.32, 69.97, 68.22, 57.82, 55.68, 53.66, 49.72, 40.46, 28.56, 15.45.

**N2-(4-(4-(2-(2-aminoethoxy)ethyl)piperazin-1-yl)phenyl)-5-chloro-N-4-(2-(isopropylsulfonyl)phenyl)pyrimidine-2,4-diamine (MKI-PEG1)**

MKI-PEG1-Boc (82.7 mg, 0.012 mmol, 1 equiv.) was dissolved with DCM (2 mL) followed by a dropwise addition of TFA (492 µL). The mixture was stirred for 2h at room temperature and then concentrated under reduced pressure. The crude mixture was purified with reverse-phase chromatography on a Biotage C18 column (acetonitrile in H_2_O, 5-95% gradient) to afford **MKI-PEG1** in quantitative yield as a yellow solid. ^1^H-NMR (500 MHz, MeOD): δ 8.43 (d, *J* = 8.5 Hz, 1H), 8.14 (s, 1H), 7.91 (dd, *J* = 7.5, 1.5 Hz, 1H), 7.66 (t, *J* = 7.0 Hz, 1H), 7.42 (dt, *J* = 7.5, 1.0 Hz, 1H), 7.37 (d, *J* = 8.5 Hz, 2H), 6.98 (d, *J* = 9.0 Hz, 2H), 3.93 (t, *J* = 4.5 Hz, 2H), 3.78 (t, *J* = 5.0 Hz, 2H), 3.73 (bs, 2H), 3.49 (t, *J* = 5.0 Hz, 2H), 3.38-3.32 (m, 1H), 3.19 (t, *J* = 5.0 Hz, 2H), 1.23 (d, *J* = 7.0 Hz, 6H). ^13^C-NMR (126 MHz, MeOD): δ 158.84, 154.53, 149.17, 145.49, 137.63, 136.09, 132.56, 131.22, 128.54, 127.07, 126.85, 125.49, 118.32, 106.76, 68.01, 65.36, 56.98, 54.75, 53.27, 47.72, 40.27, 15.38.

**N-(2-(2-(4-(4-((5-chloro-4-((2-(isopropylsulfonyl)phenyl)amino)pyrimidin-2-yl)amino)phenyl)piperazin-1-yl)ethoxy)ethyl)acrylamide (MKI-AA3)**

This compound was synthesized with the same procedure as MKI-AA, using acrylic acid (4.3 μL, 0.068 mmol, 1.2 equiv.), T3P (61 mg, 0.097 mmol, 1.7 equiv.), DIPEA (29 μL, 0.17 mmol, 3 equiv.), MKI-PEG1 (33 mg, 0.057 mmol, 1 equiv.) and DMF (2 mL). The preparative TLC was run with MeOH/DCM 5:95 to yield **MKI-AA3** as a yellow solid (3.7 mg, 5.9 μmol, 10%). ^1^H-NMR (500 MHz, CDCl_3_): δ 9.61 (s, 1H), 8.59 (d, *J* = 8.5 Hz, 1H), 8.10 (s, 1H), 7.89 (dd, *J* = 7.5, 1.5 Hz, 1H), 7.56 (dt, *J* = 8.0, 1.5 Hz, 1H), 7.49 (d, *J* = 9.0 Hz, 2H), 7.23 (dt, *J* = 7.5, 1.5 Hz, 1H), 6.95 (bs, 1H), 6.89 (d, *J* = 8.5 Hz, 2H), 6.30 (dd, *J* = 17.0, 1.5 Hz, 1H),6.18-6.12 (m, 1H), 5.62 (dd, *J* = 10.0, 1.5 Hz, 1H), 3.69-3.66 (m, 2H), 3.61-3.59 (m, 2H), 3.57-3.53 (m, 2H), 3.26-3.20 (m, 4H), 2.81-2.71 (m, 6H), 1.31 (d, *J* = 7.0 Hz, 6H). ^13^C-NMR (126 MHz, CDCl_3_): δ 168.57, 165.73, 158.23, 155.52, 155.32, 138.63, 134.58, 132.02, 131.38, 131.06, 126.53, 124.54, 123.55, 123.18, 122.23, 117.04, 106.01, 69.69, 57.82, 55.74, 53.67, 49.53, 39.26, 29.85, 15.51. HRMS (ESI+) m/z calculated for C_30_H_38_ClN_7_O_4_S [M+H]^+^: 628.2467; found: 628.2490.

**^
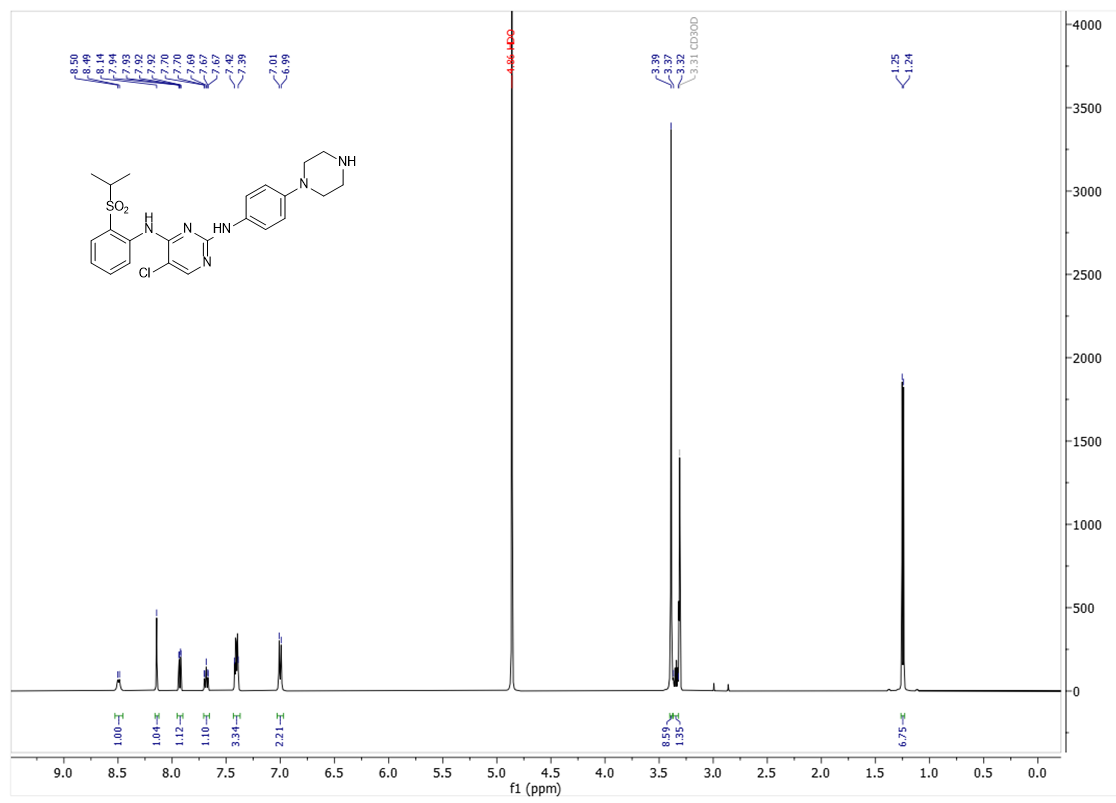
1^H-NMR for MKI-NH (500 MHz, MeOD)**

**^13^C-NMR for MKI-NH (126 MHz, MeOD)**

**^
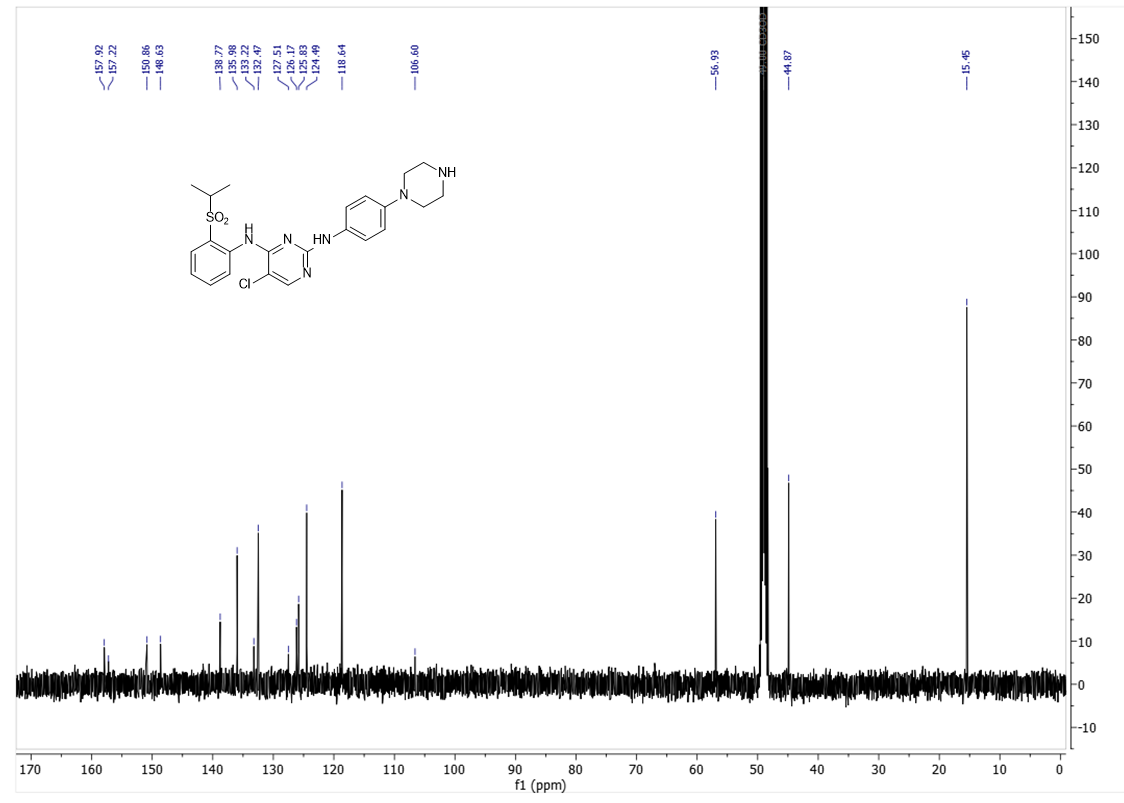
^**

**^
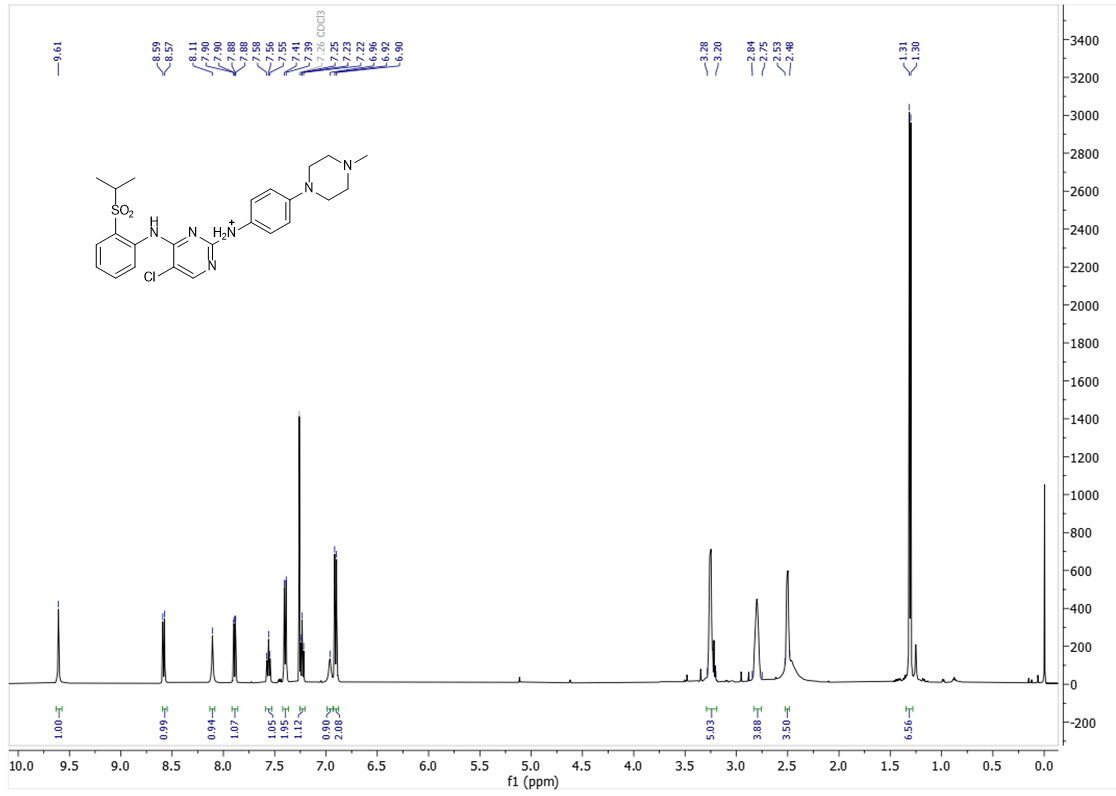
1^H-NMR for TL13-87 (500 MHz, CDCl_3_)**

**^
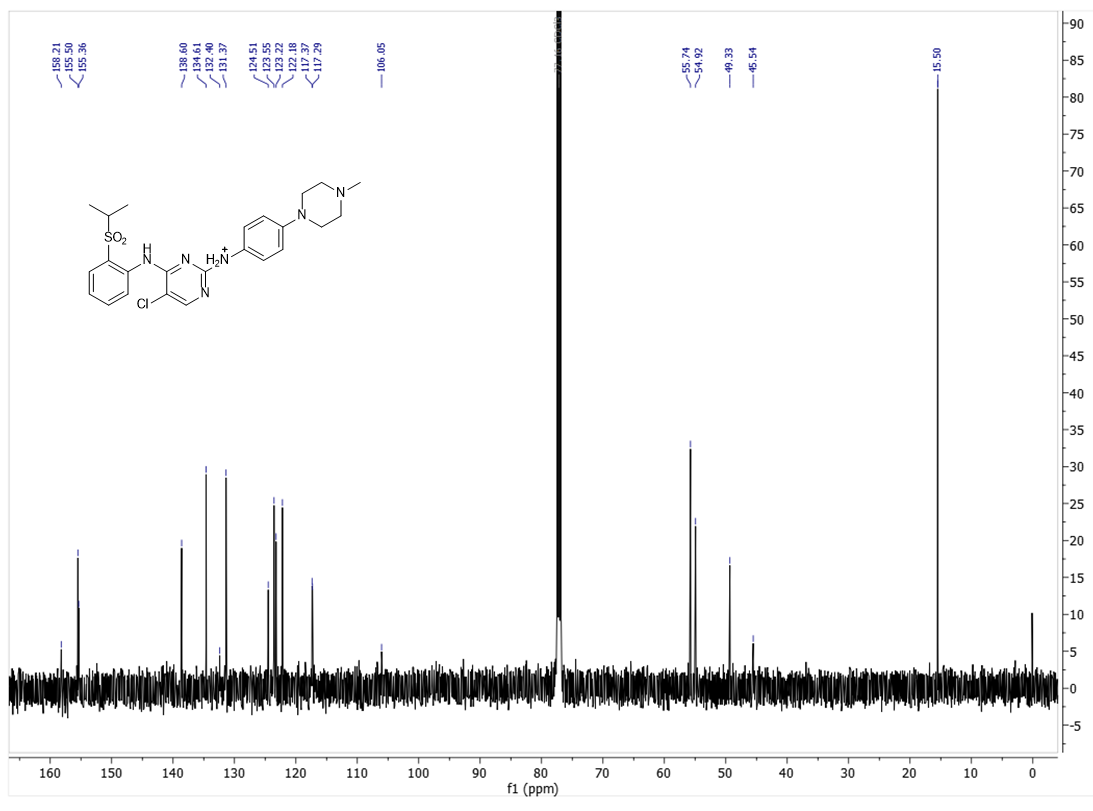
13^C-NMR for TL13-87 (126 MHz, CDCl_3_)**

**^1^H-NMR for MKI-AA (500 MHz, CDCl_3_)
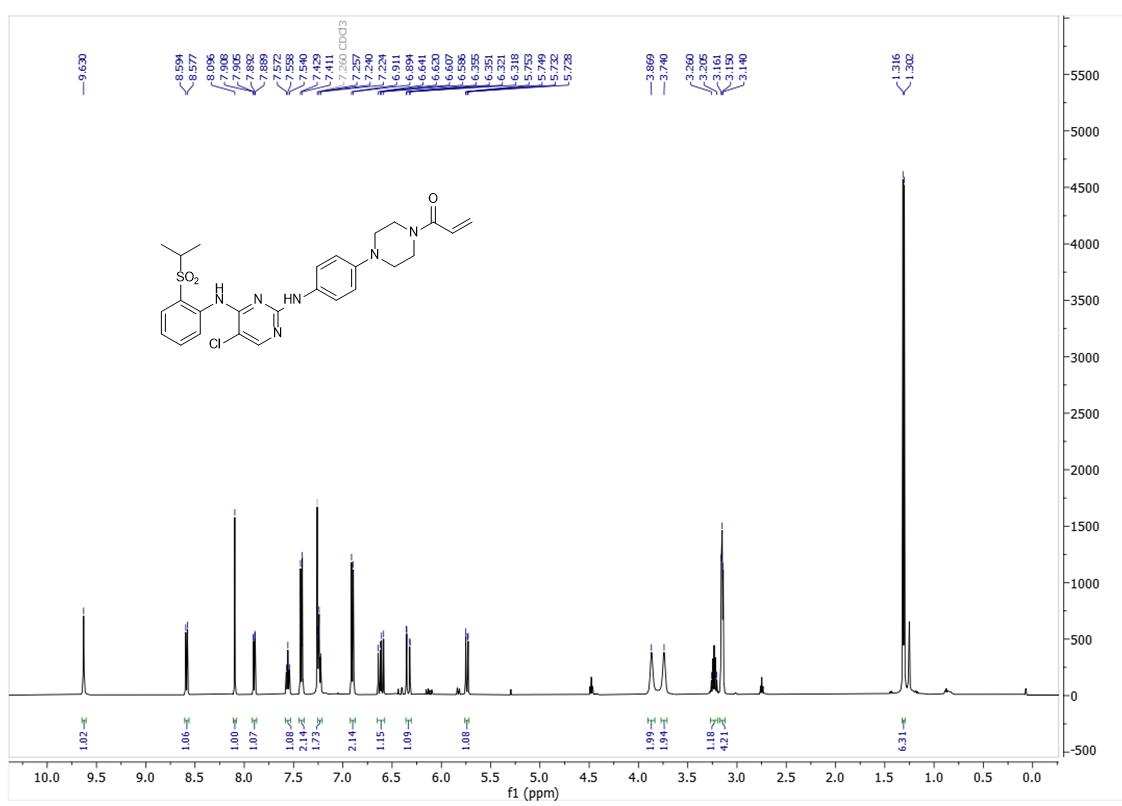
**

**^13^C-NMR for MKI-AA (126 MHz, CDCl_3_)**

**^
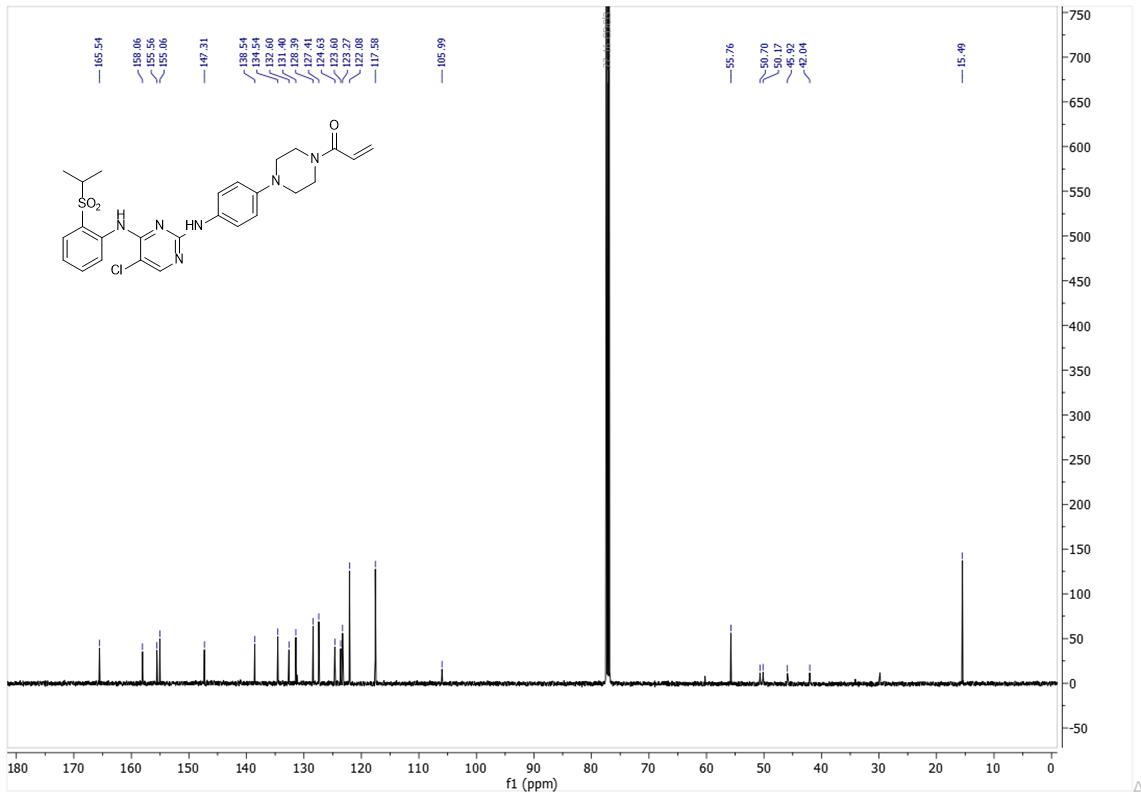
^**

**^1^H-NMR for MKI-CA (500 MHz, CDCl_3_)
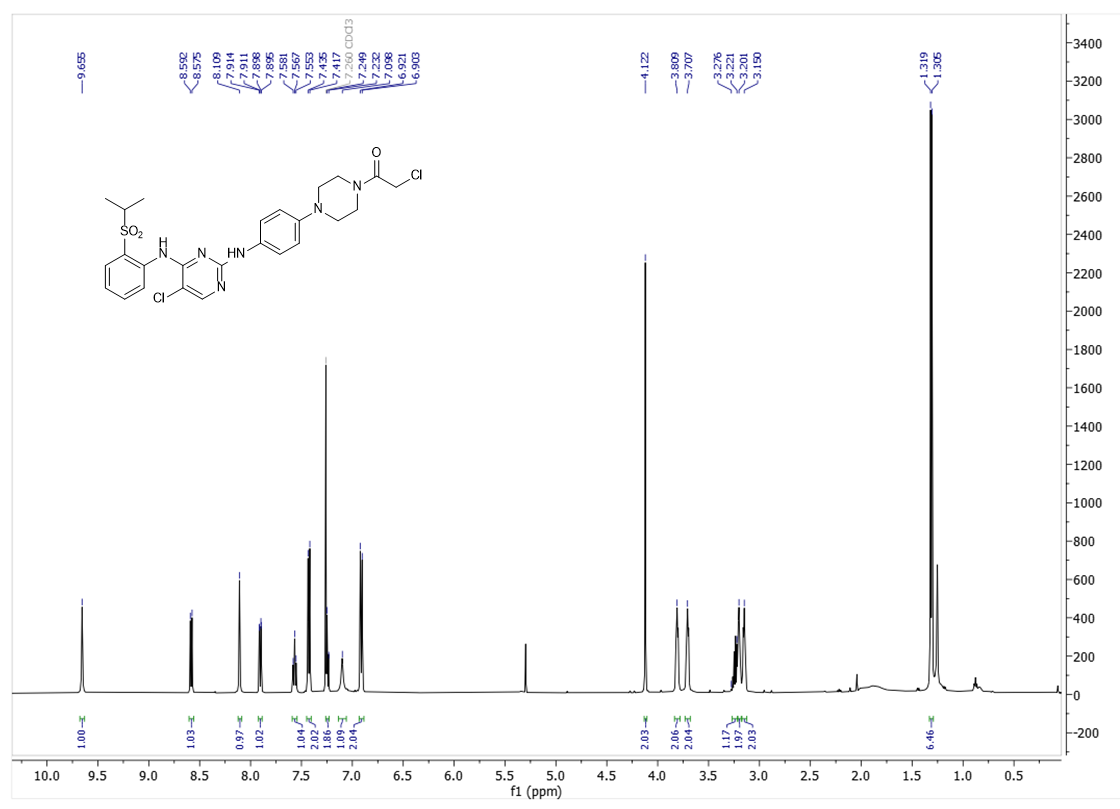
**

**^13^C-NMR for MKI-CA (126 MHz, CDCl_3_)**

**
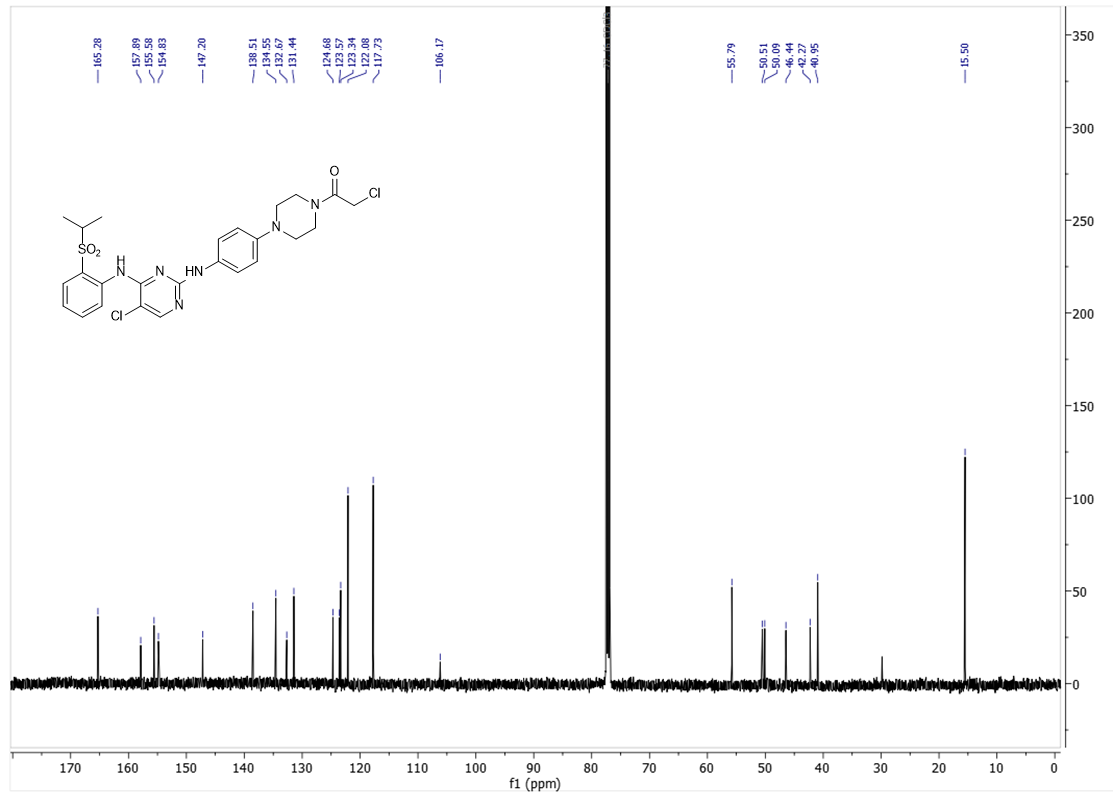
**

**^
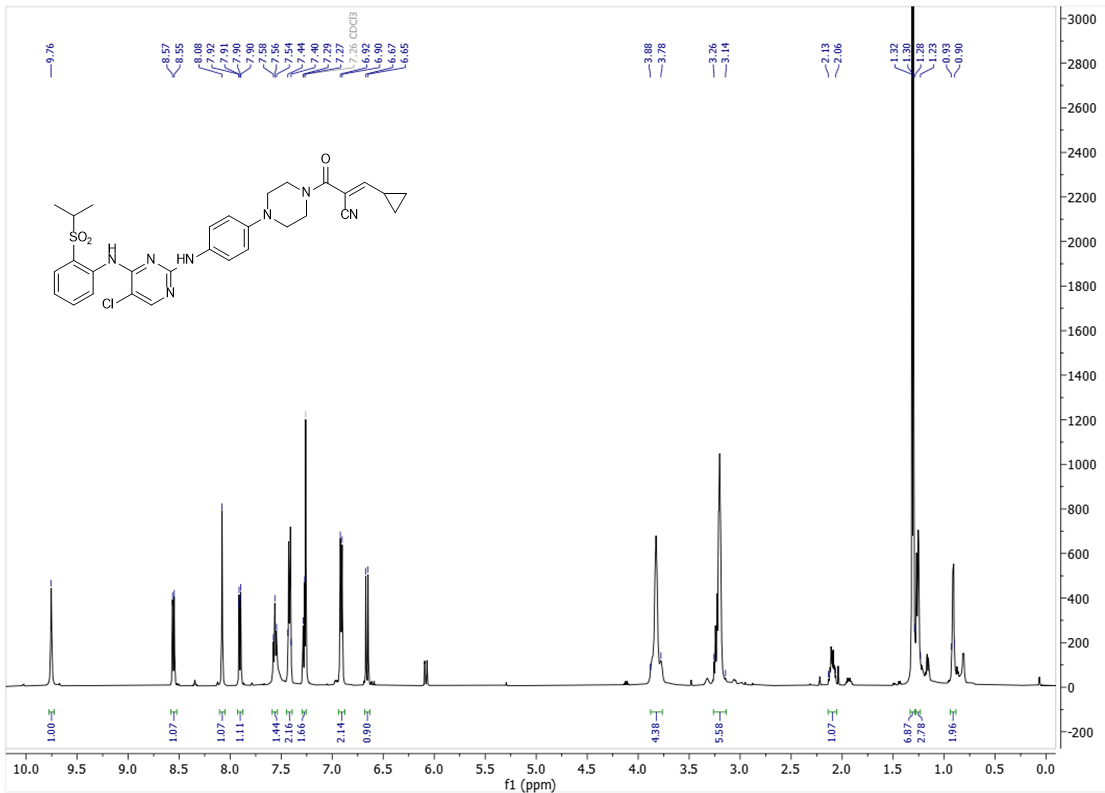
1^H-NMR for MKI-CPA (500 MHz, CDCl_3_)**

**^13^C-NMR for MKI-CPA (126 MHz, CDCl_3_)**

**^
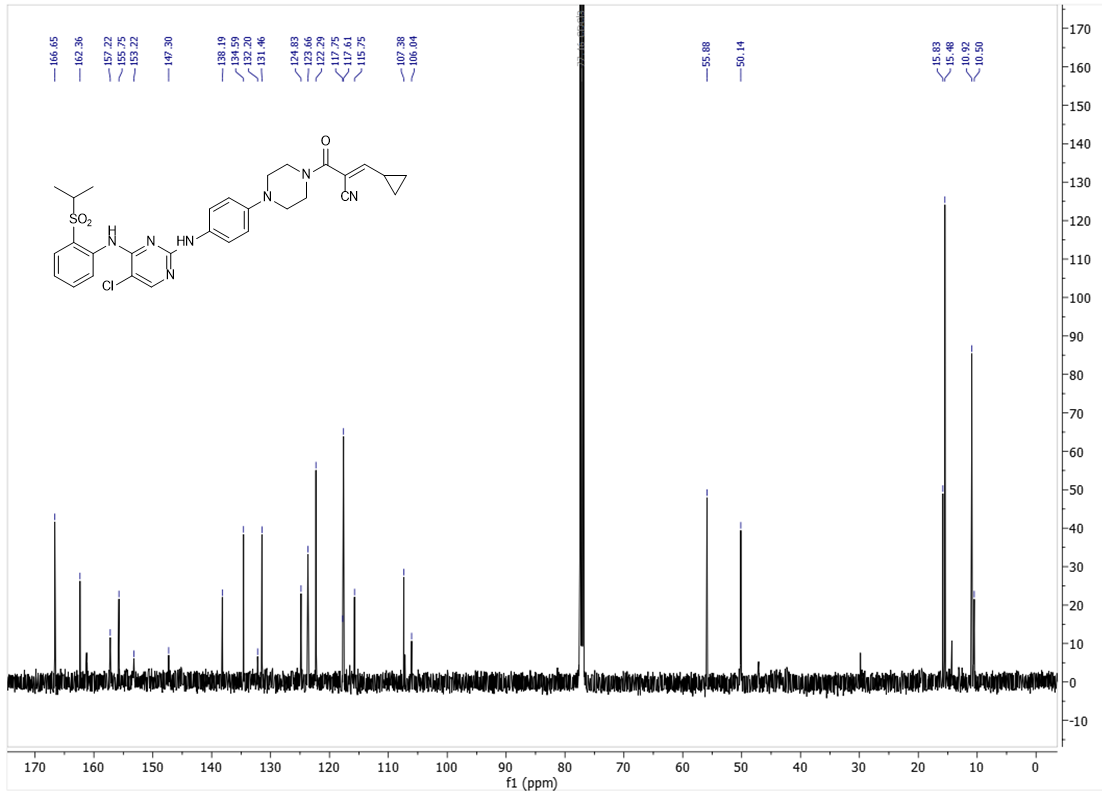
^**

**^
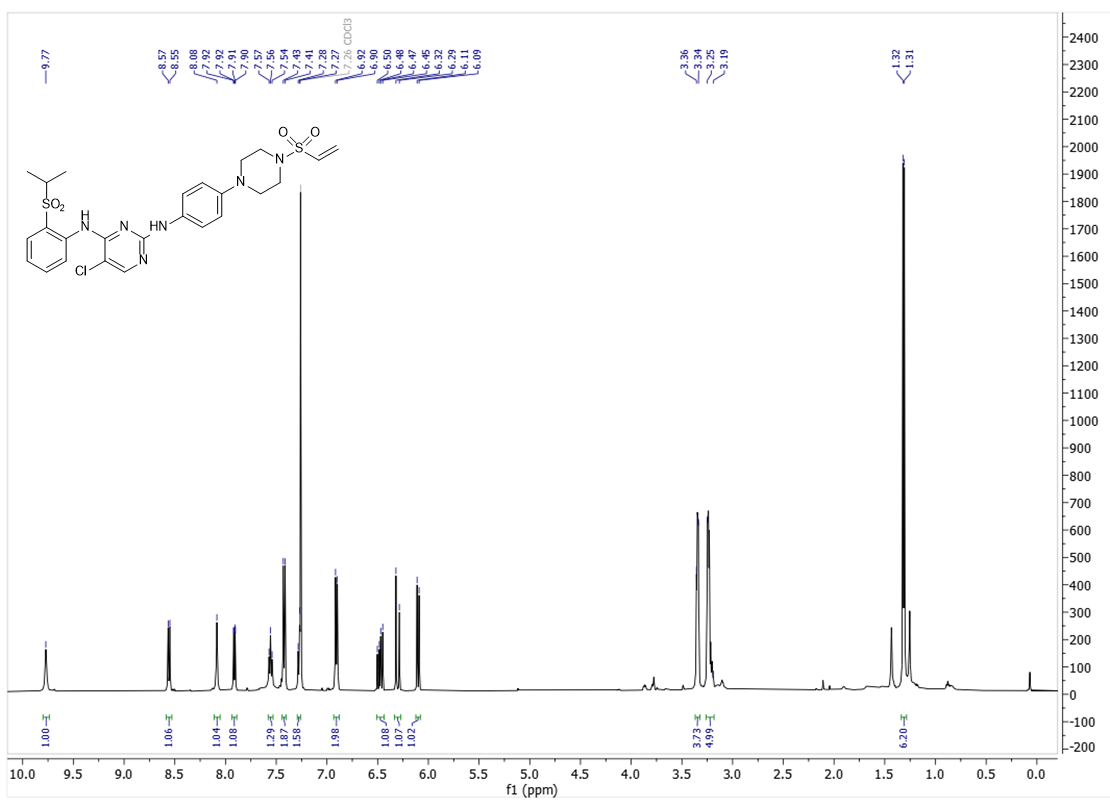
1^H-NMR for MKI-VSA (500 MHz, CDCl_3_)**

**^13^C-NMR for MKI-VSA (126 MHz, CDCl_3_)**

**^
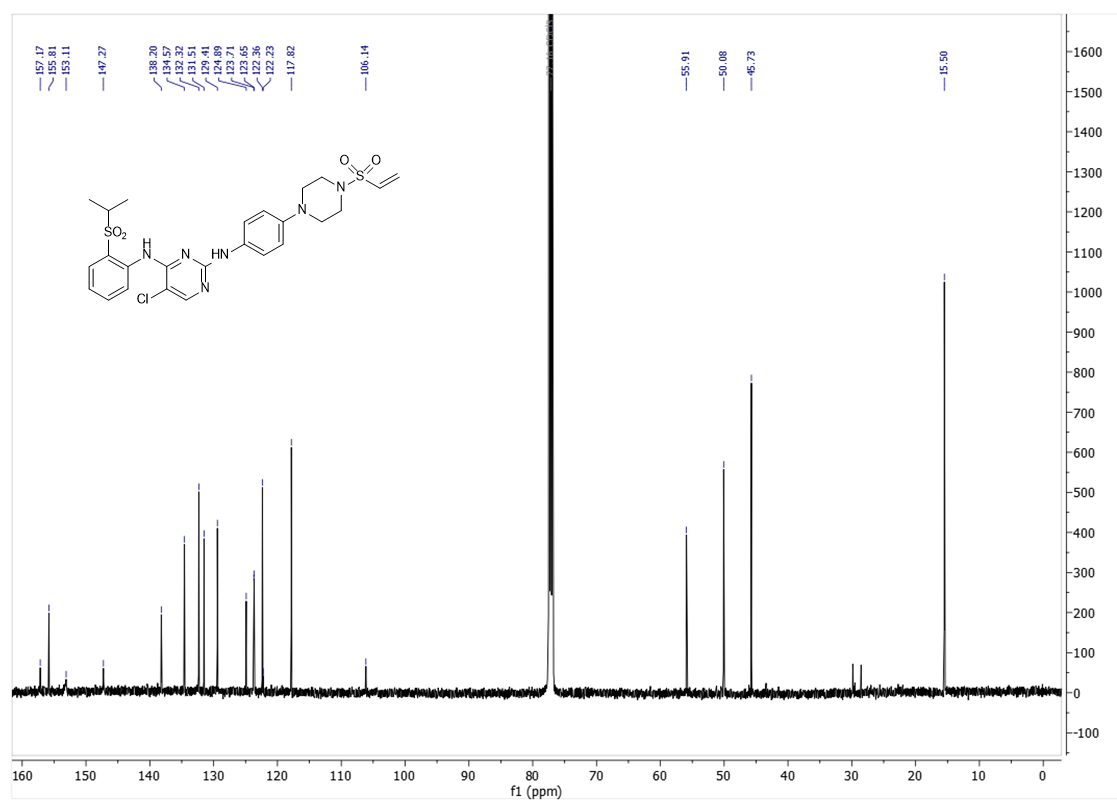
^**

**^1^H-NMR for MKI-PA (500 MHz, CDCl_3_)^
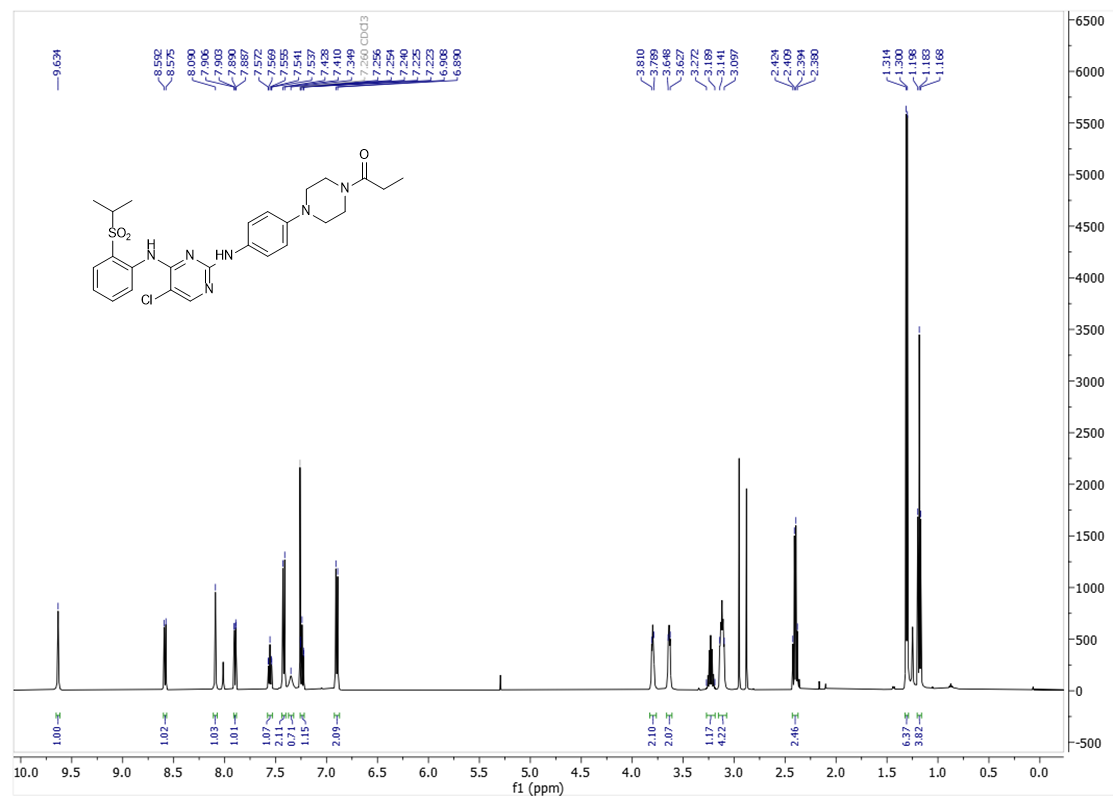
^**

**^13^C-NMR for MKI-PA (126 MHz, CDCl_3_)**

**^
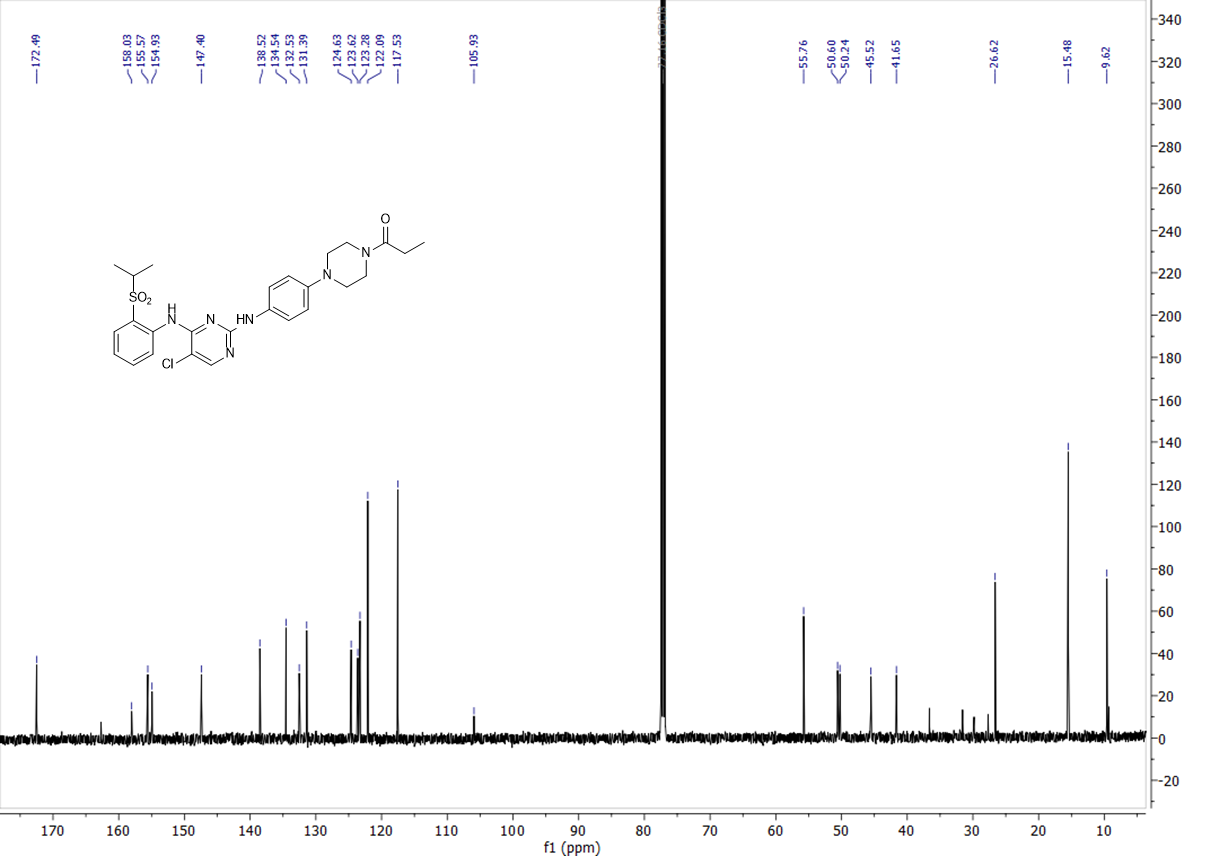
^**

**^1^H-NMR for MKI-A-Boc (500 MHz, CDCl_3_)
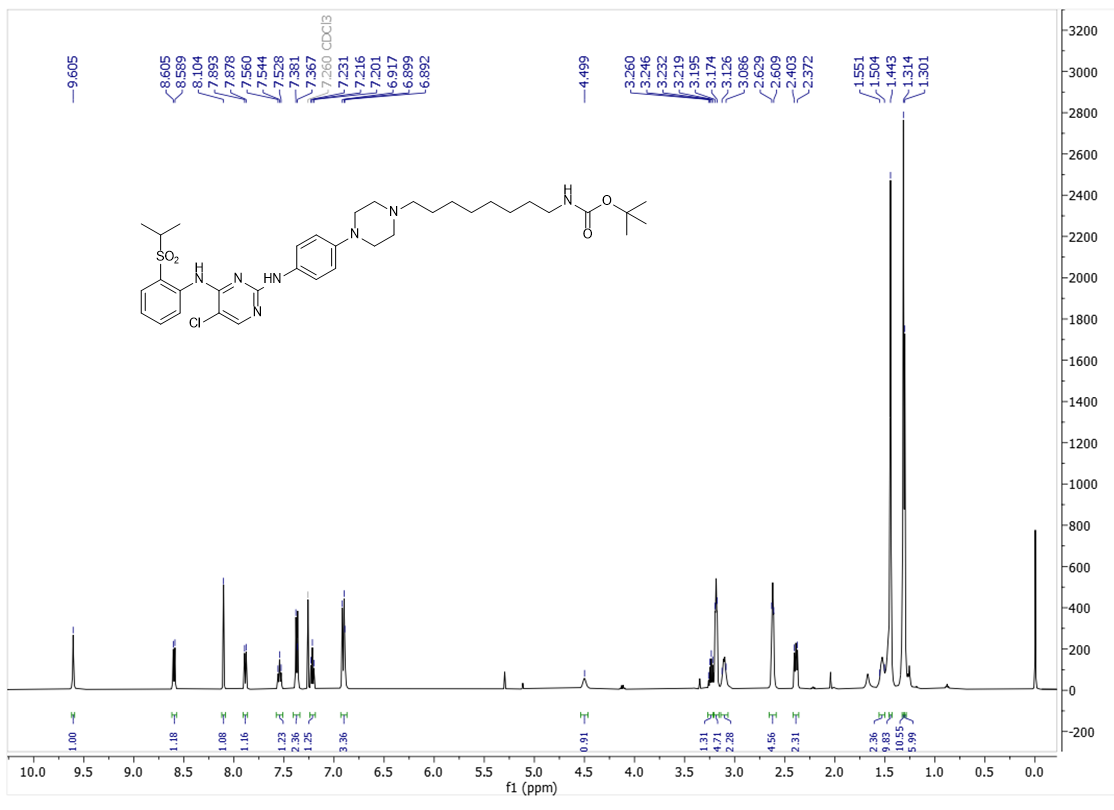
**

**^13^C-NMR for MKI-A-Boc (126 MHz, CDCl_3_)**

**
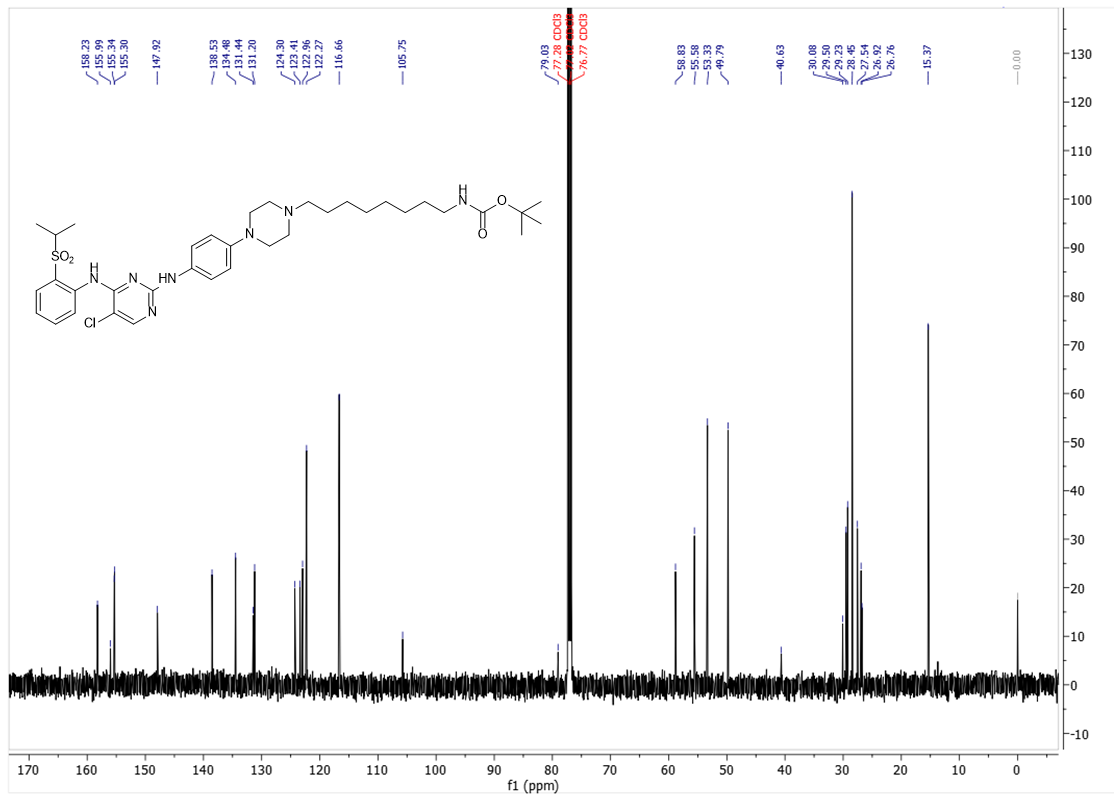
**

**^1^H-NMR for MKI-A (500 MHz, MeOD)
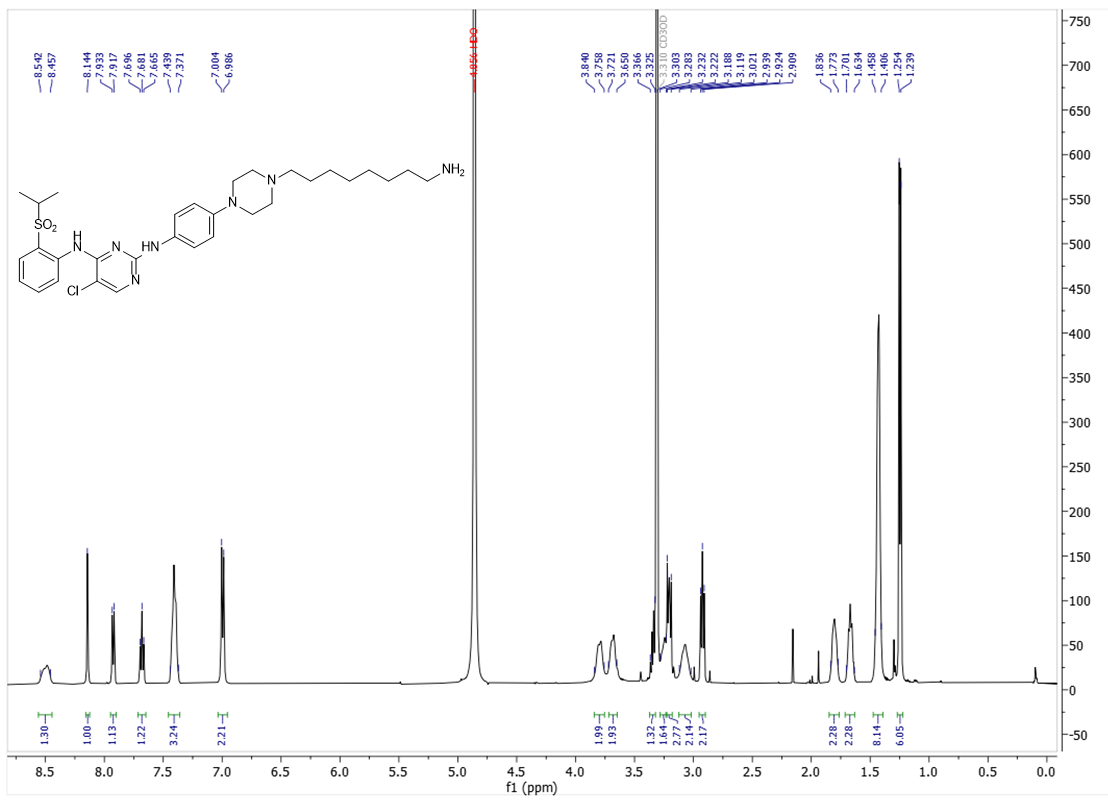
**

**^13^C-NMR for MKI-A (126 MHz, MeOD)**

**^
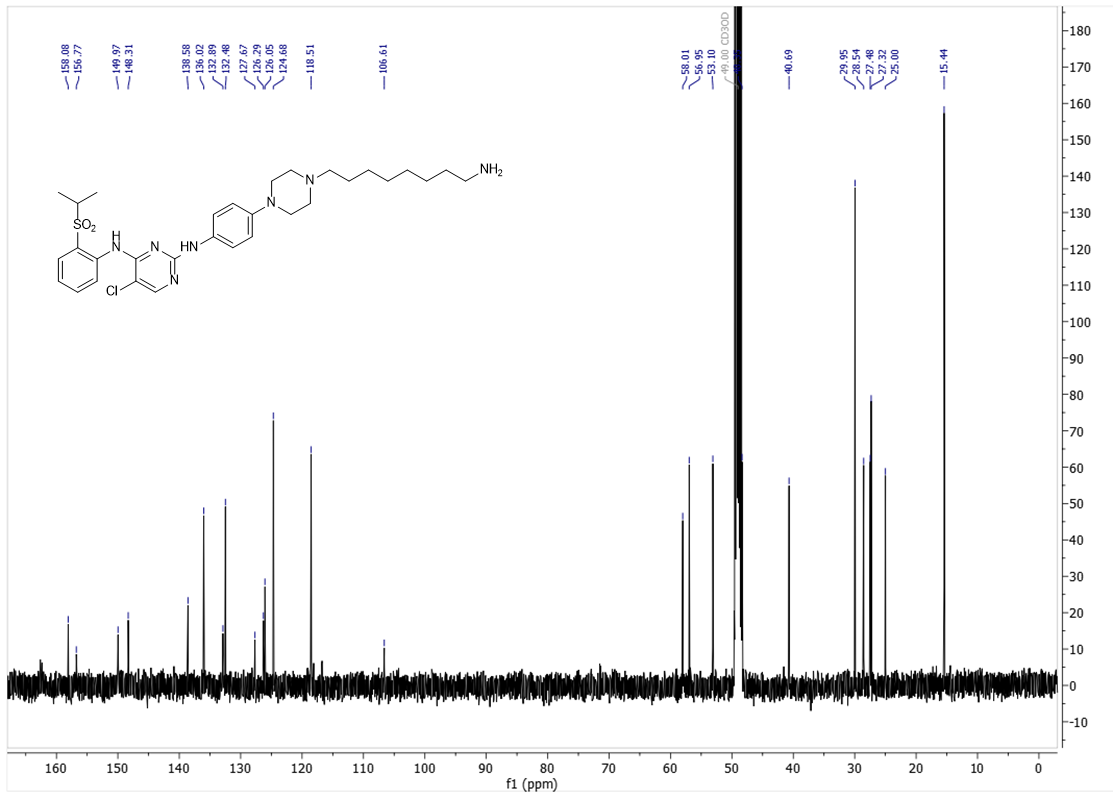
^**

**^1^H-NMR for MKI-Gly-Boc (500 MHz, CDCl_3_)
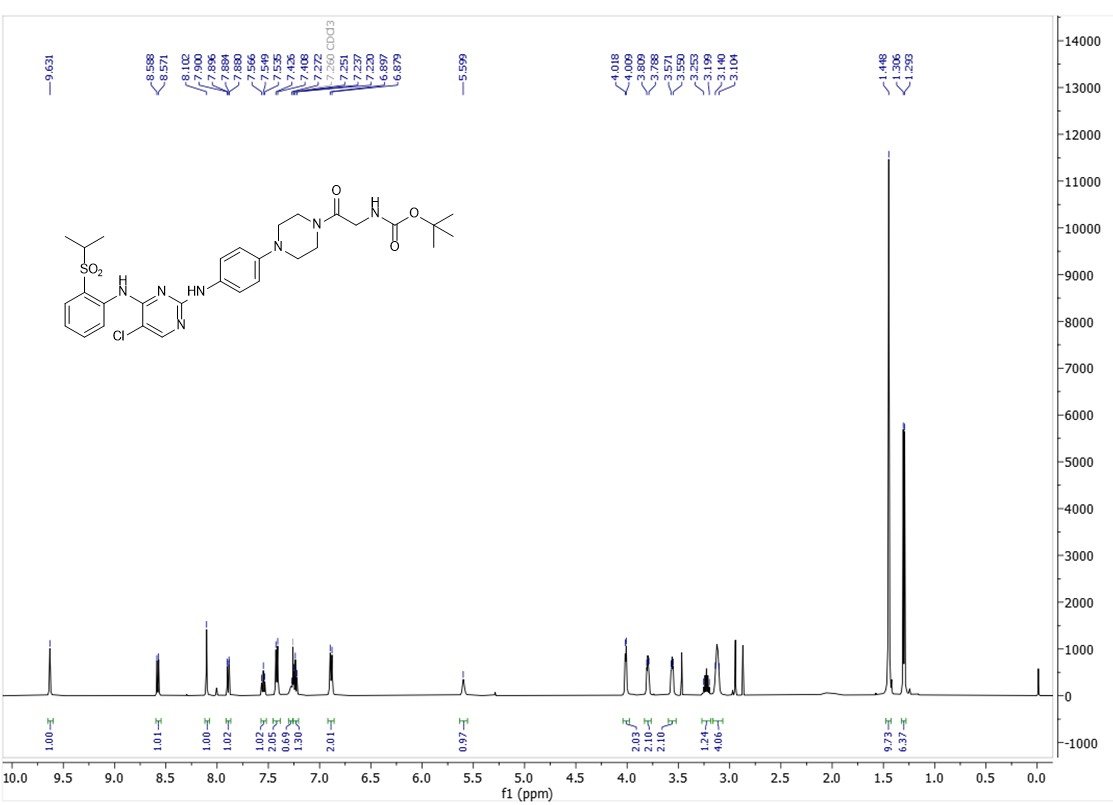
**

**^13^C-NMR for MKI-Gly-Boc (126 MHz, CDCl_3_)**

**
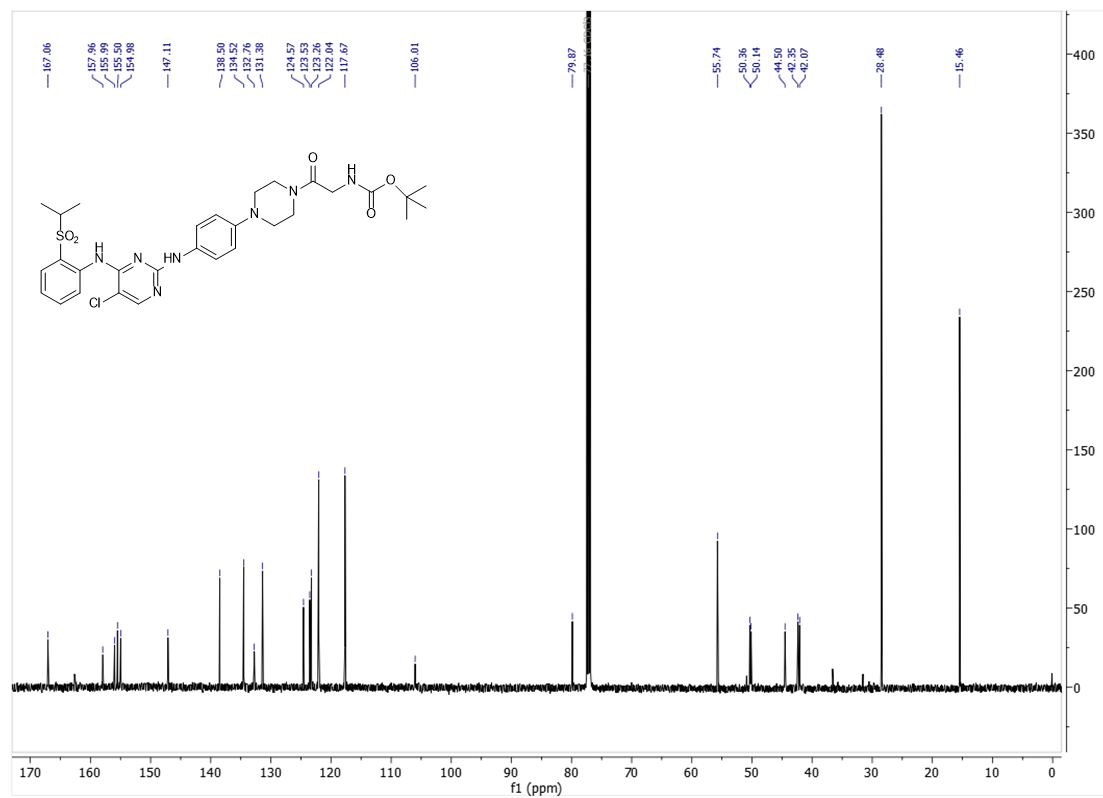
**

**^1^H-NMR for MKI-Gly (500 MHz, MeOD)
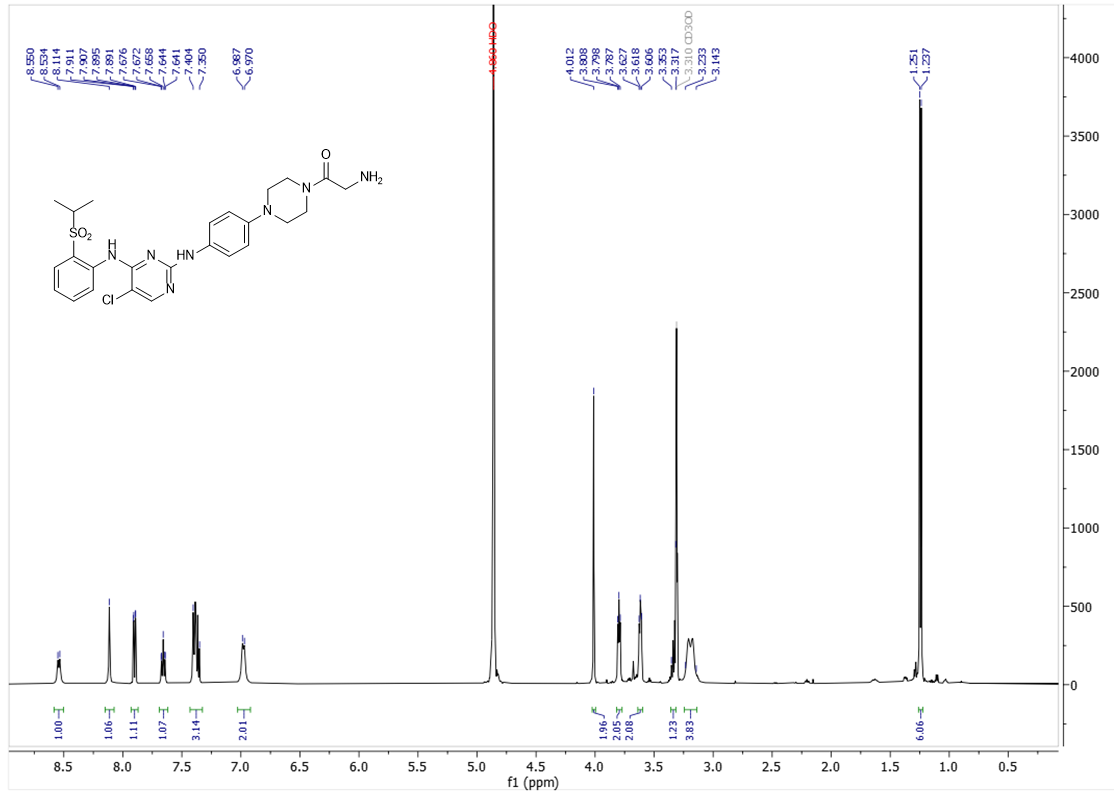
**

**^13^C-NMR for MKI-Gly (126 MHz, MeOD)**

**
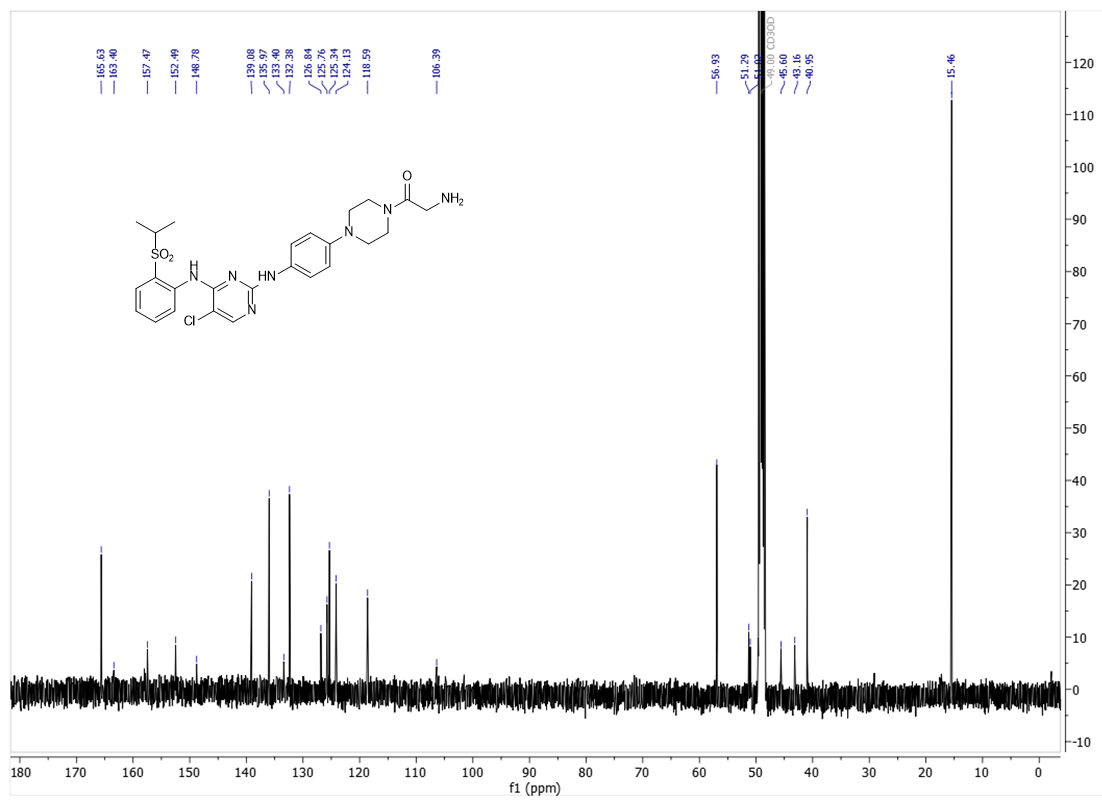
**

**^1^H-NMR for MKI-AA2 (500 MHz, CDCl_3_)^
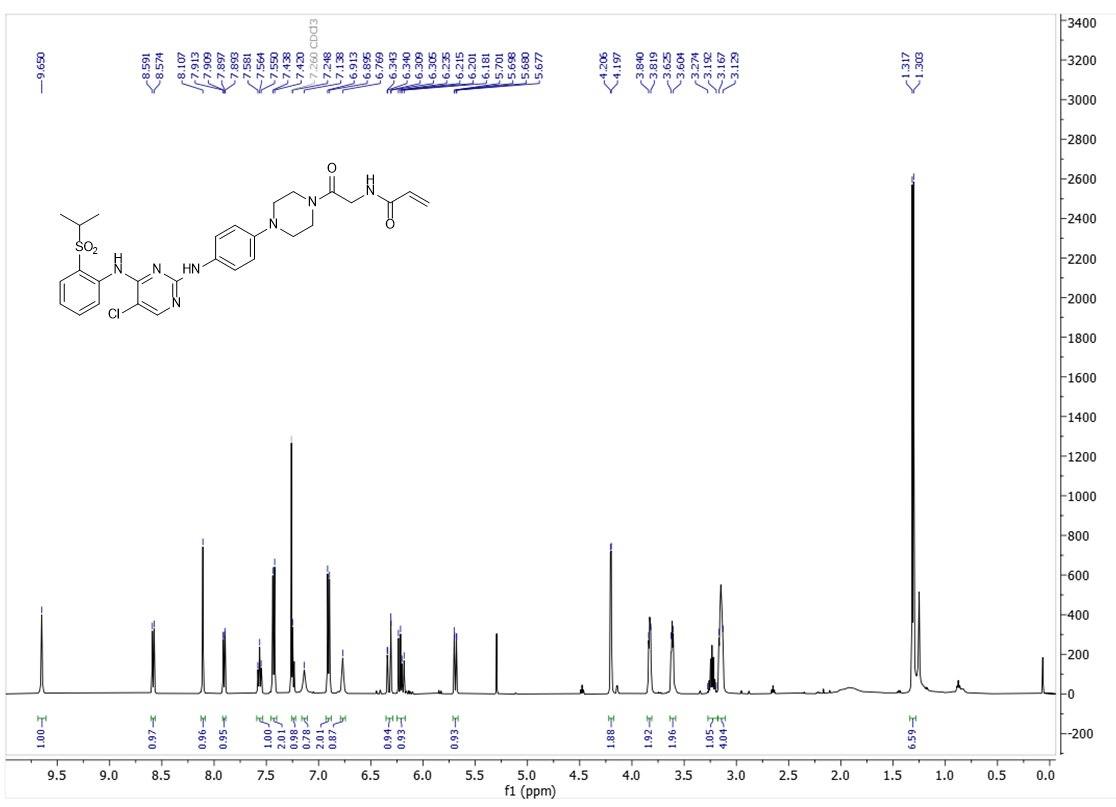
^**

**^13^C-NMR for MKI-AA2 (126 MHz, CDCl_3_)**

**^
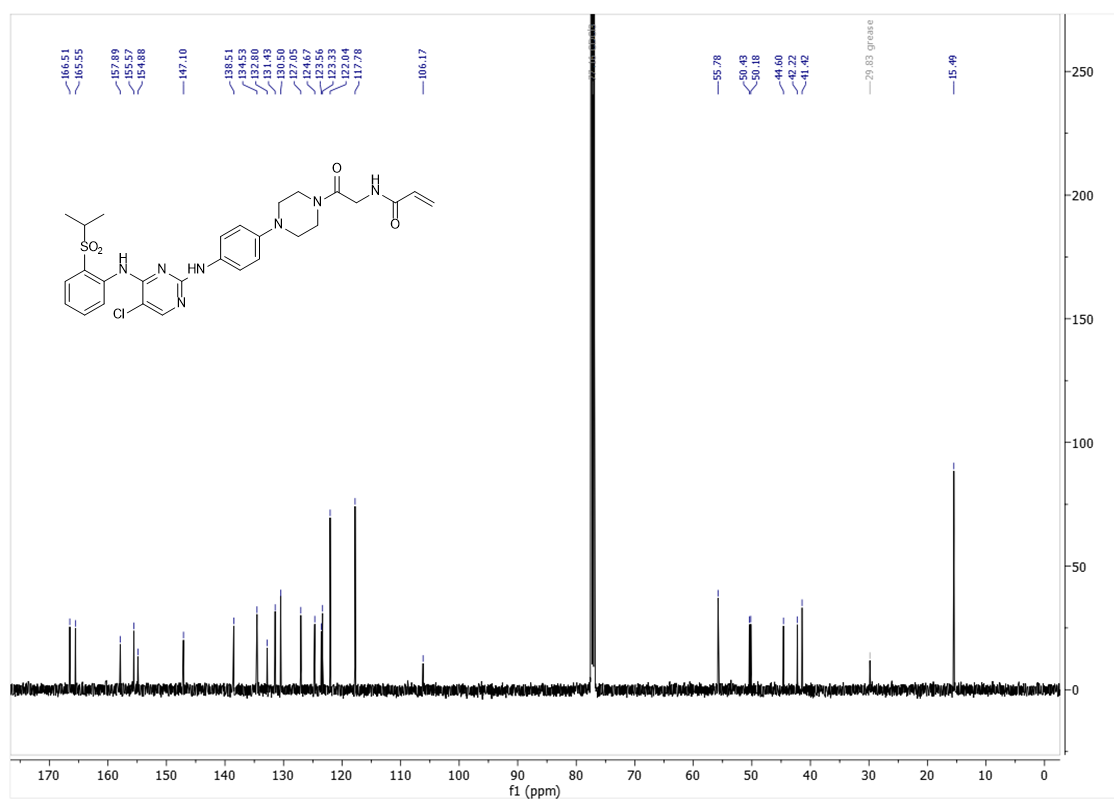
^**

**^1^H-NMR for MKI-PEG1-Boc (500 MHz, CDCl_3_)
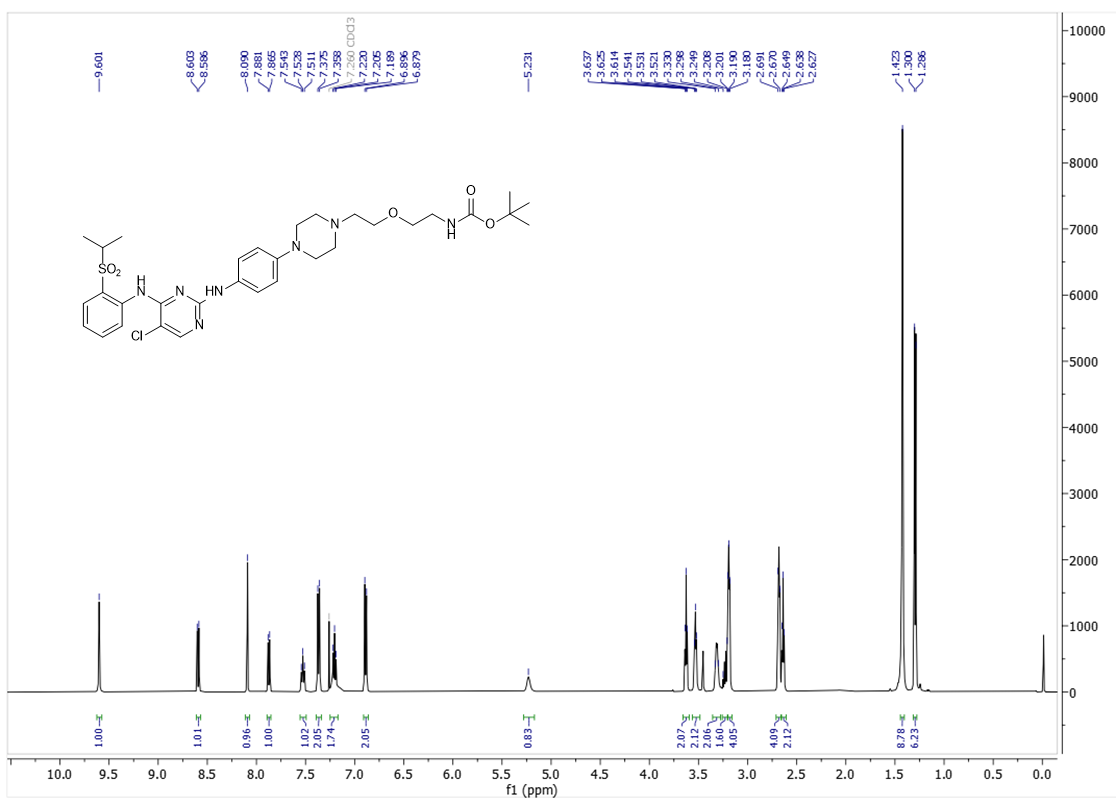
**

**^13^C-NMR for MKI-PEG1-Boc (126 MHz, CDCl_3_)**

**
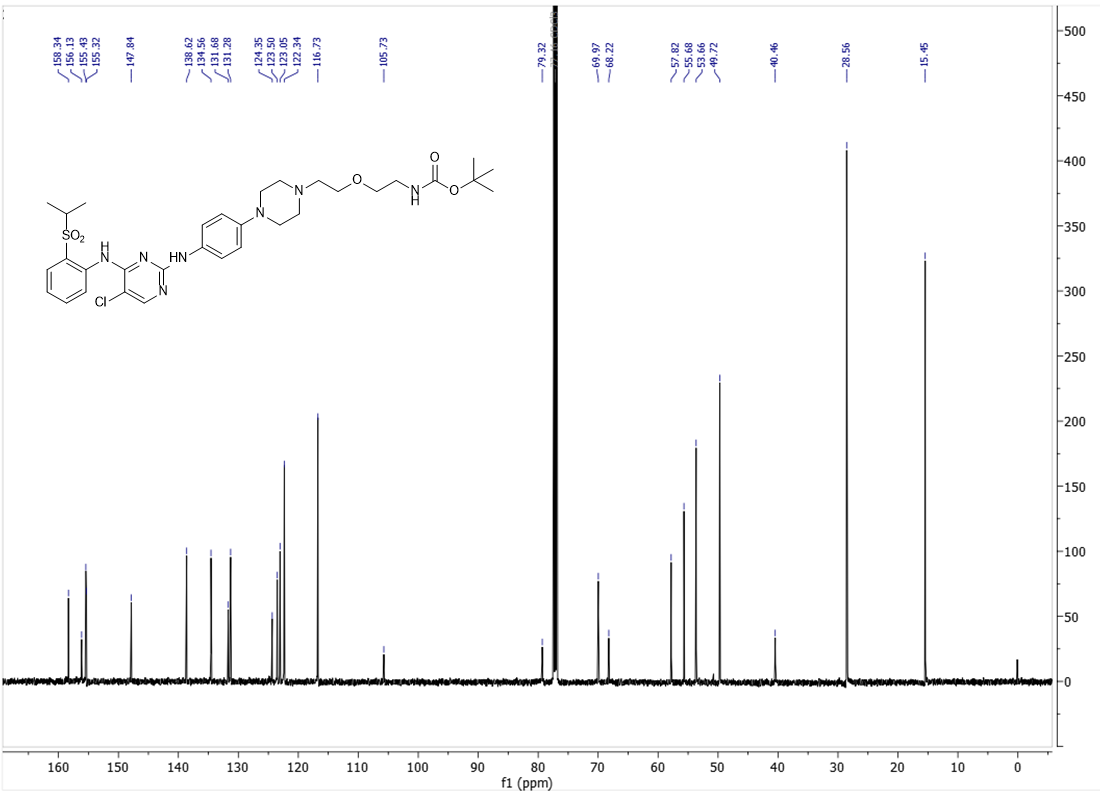
**

**^1^H-NMR for MKI-PEG1 (500 MHz, MeOD)^
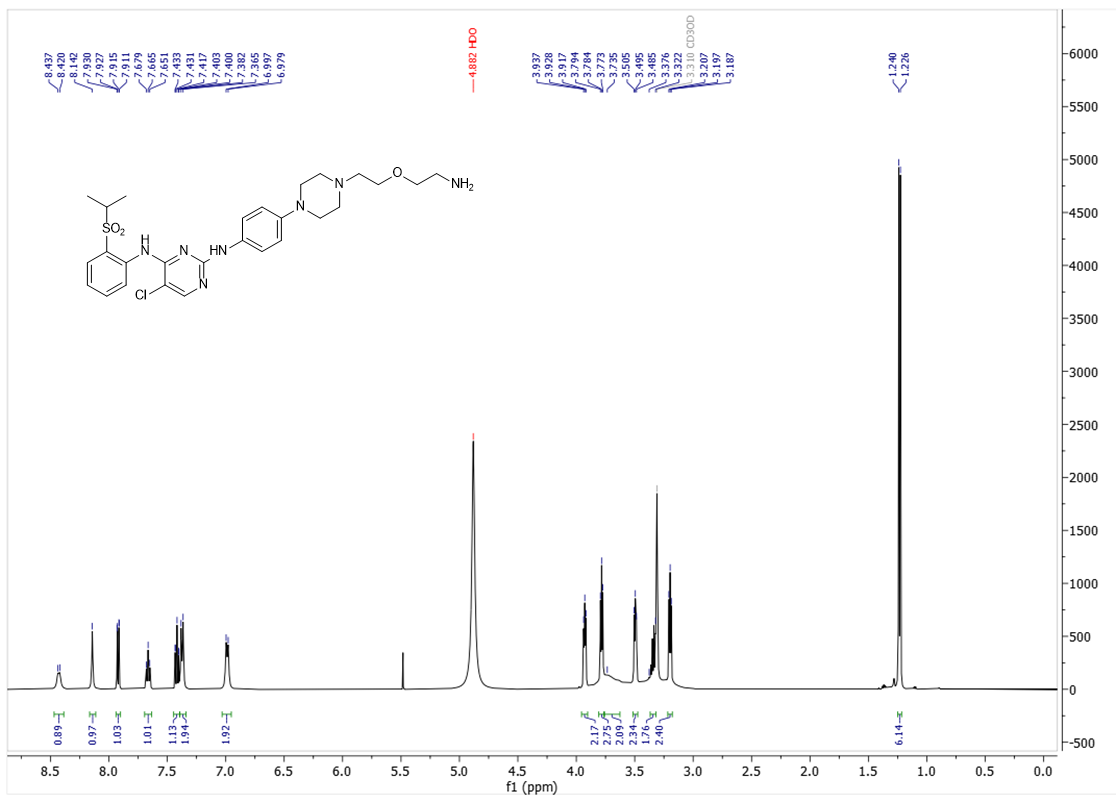
^**

**^13^C-NMR for MKI-PEG1 (126 MHz, MeOD)**

**^
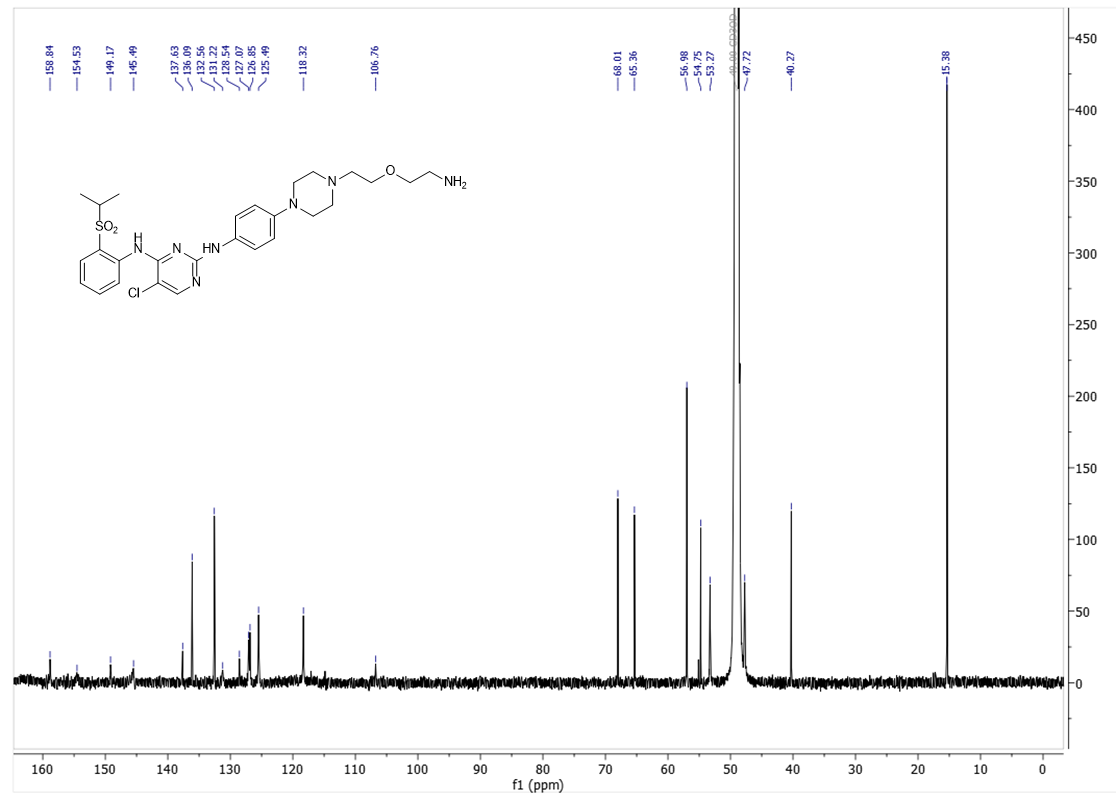
^**

**^1^H-NMR for MKI-AA3 (500 MHz, CDCl_3_)
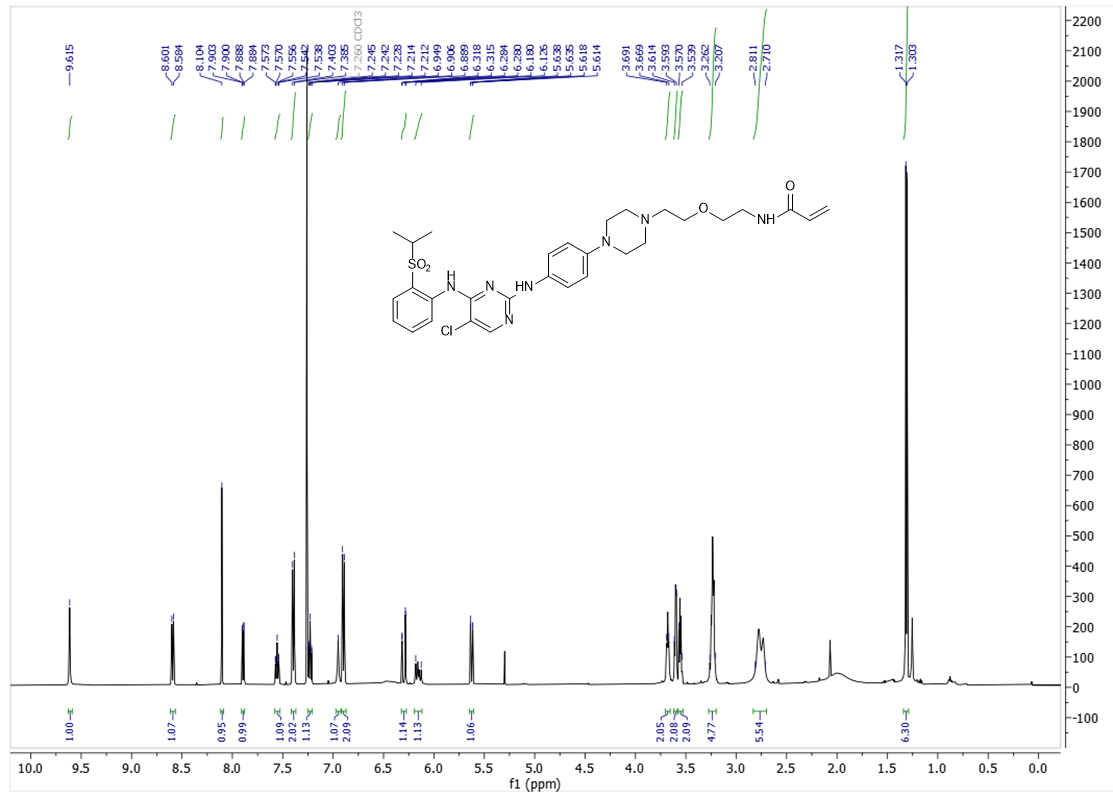
**

**^13^C-NMR for MKI-AA3 (126 MHz, CDCl_3_)
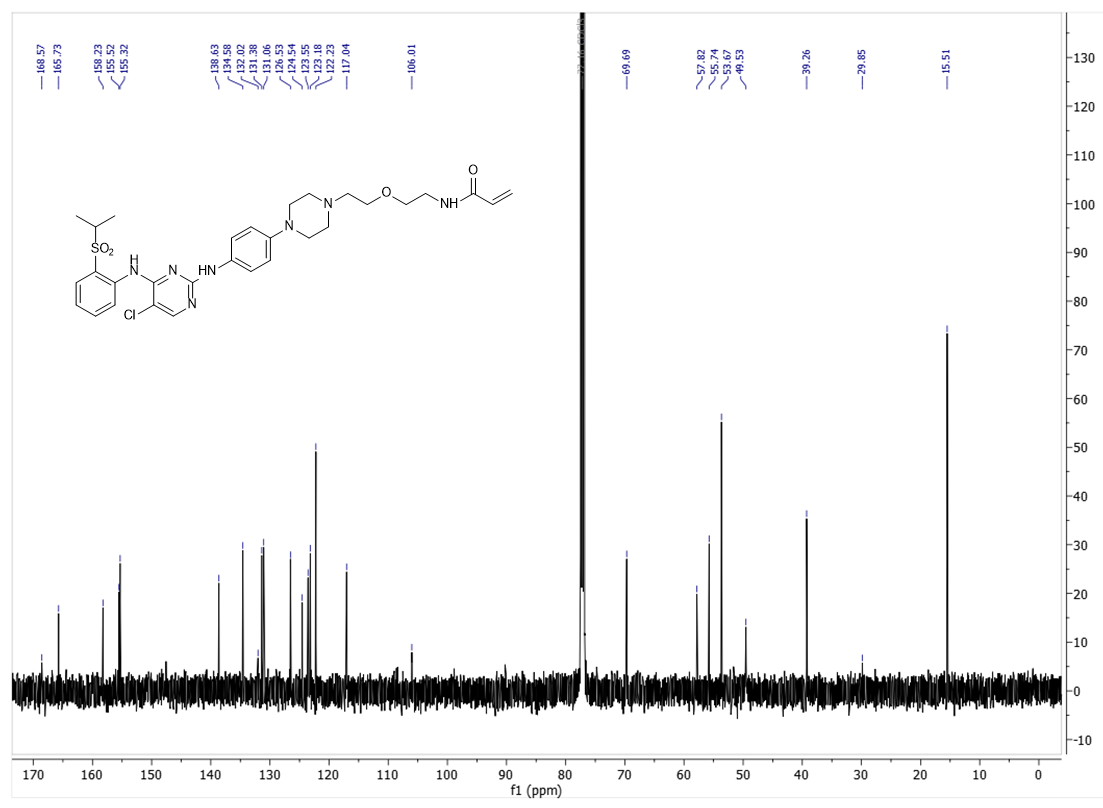
**

#

**4. Supplementary References**

1. H. T. Huang, D. Dobrovolsky, J. Paulk, G. Yang, E. L. Weisberg, Z. M. Doctor, D. L. Buckley, J. H. Cho, E. Ko, J. Jang, K. Shi, H. G. Choi, J. D. Griffin, Y. Li, S. P. Treon, E. S. Fischer, J. E. Bradner, L. Tan, N. S. Gray, "A Chemoproteomic Approach to Query the Degradable Kinome Using a Multi-kinase Degrader," *Cell Chem Biol* 25 (2018): 88, https://doi.org/10.1016/j.chembiol.2017.10.005.
